# Self-assembled nanoparticles from natural herbs alleviate osteoarthritis by targeting retinol metabolism and inhibiting chondrocyte ferroptosis

**DOI:** 10.1016/j.mtbio.2026.103250

**Published:** 2026-05-17

**Authors:** Xuefeng Li, Yuhang Wang, Xucheng Wang, Wenzhe Chen, Ling Jin, Qinwen Ge, Jingyuan Wen, Pinger Wang, Wenhua Yuan, Yimin Yang, Luwei Xiao, Jiali Chen, Di Chen, Songfeng Hu, Hongting Jin

**Affiliations:** a Institute of Orthopaedics and Traumatology, The First Affiliated Hospital of Zhejiang Chinese Medical University (Zhejiang Provincial Hospital of Chinese Medicine), Hangzhou, China; b The First College of Clinical Medicine, Zhejiang Chinese Medical University, Hangzhou, China; c Faculty of Pharmaceutical Sciences, Shenzhen Institute of Advanced Technology, Shenzhen, China; d Shaoxing Hospital of Traditional Chinese Medicine, Shaoxing, China

**Keywords:** Osteoarthritis, Self-assembled nanoparticles, Hydrogel, Retinol metabolism, Ferroptosis

## Abstract

Osteoarthritis (OA), a prevalent degenerative joint disease, currently lacks effective therapies. Self-assembled nanomedicines derived from herbs create innovative pharmaceutical formulations, and offer promising therapeutic strategies for OA treatment. Considering the treatment of Shen-Sui-An-Kang (SSAK) decoction in clinical OA symptoms, here we isolated the nanoparticles from SSAK decoction, and then investigated their effects on OA treatment. By using gradient centrifugation and dialysis, self-assembled nanoparticles of SSAK (N-SSAK) were isolated based on the hydrophobic interactions, hydrogen bonding, and π-π stacking. After determining the physical characteristics of N-SSAK, we investigated the therapeutic effect of N-SSAK *in vivo* and *in vitro*, and found that they alleviated cartilage degeneration, maintained the homeostasis of cartilage matrix, and reduced pain sensitivity. Mechanistically, we demonstrated that N-SSAK exerted their effects by modulating retinol metabolism and inhibiting lipid peroxidation in OA chondrocytes. To improve the sustained release and targeted delivery of N-SSAK, we developed an injectable hydrogel-based delivery system (HA-MIX@N-SSAK). Compared to N-SSAK oral administration, HA-MIX@N-SSAK showed better efficacy on cartilage protection and slowing OA progression. Overall, our findings indicate that N-SSAK suppress the progression of OA by regulating retinol metabolism and inhibiting chondrocyte ferroptosis, implying that HA-MIX@N-SSAK may be a novel strategy for clinical OA treatment.

## Graphical abstract

**Figure:**
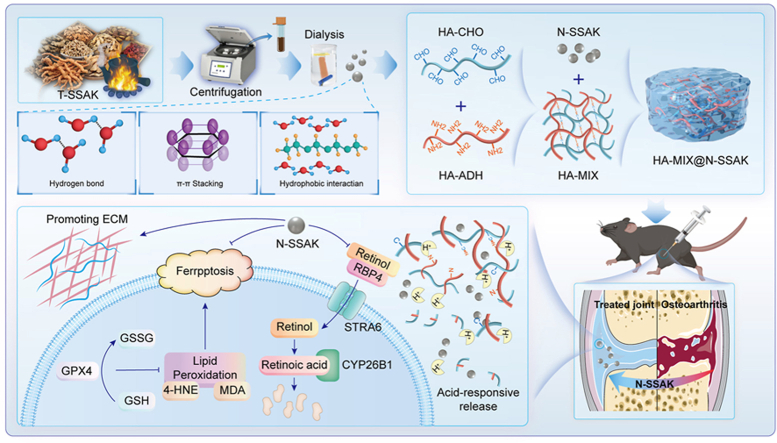

## 1 Introduction

Osteoarthritis (OA) is the most prevalent degenerative musculoskeletal disease, affecting over 500 million people worldwide [1]. Its pathological features primarily include progressive articular cartilage degradation, abnormal subchondral bone remodeling, synovial inflammation, and compromised periarticular soft tissue stability [2]. Clinically manifesting as joint pain, stiffness, swelling, and functional impairment, OA has an increasingly high prevalence with the acceleration of population aging. Regrettably, effective clinical management strategies for OA remain limited. Current pharmacological interventions primarily focus on managing clinical symptoms, yet long-term administration carries risks of cardiovascular and gastrointestinal adverse effects while failing to reverse structural cartilage damage [3]. Although joint replacement serves as an effective treatment for end-stage OA, it presents challenges such as postoperative infections, substantial financial burden, and limited suitability for early-stage patients [4,5]. Therefore, developing novel therapeutic strategies capable of effectively delaying OA progression represents an urgent clinical need.

Traditional Chinese medicine (TCM), with its long history, is gaining global attention for its multi-component and multi-target approach to OA management [6]. The fundamental TCM theory of “kidney governing bones and generating marrow” provides the theoretical foundation for OA prevention and treatment [7]. The natural herb-based Shen-Sui-An-Kang decoction (T-SSAK), as a representative formula for nourishing kidney and strengthening bones, has demonstrated promising therapeutic potential in OA according to preliminary studies [8]. Phytochemical analyses have revealed that T-SSAK contains various bioactive compounds, including alkaloids, terpenoids, and flavonoids, which collectively contribute to its multiple pharmacological effects encompassing analgesia, anti-inflammation, and antioxidant activities [[9], [10], [11]]. Nevertheless, the clinical translation of traditional decoctions faces significant challenges due to poor water solubility, low bioavailability, insufficient targeting capability, and short duration of action [12]. In recent years, self-assembled nanoparticles of Chinese herbal medicine have emerged as a novel research direction. Through hydrophobic interactions, hydrogen bonding, and π-π stacking forces, herbal components spontaneously form nanostructures during the decoction process [13]. This self-assembly enhances drug solubility, absorption efficiency, and overall therapeutic efficacy [14]. For example, the nanoparticles from QY305 (N-QY305) show improved efficacy and reduced toxicity compared to the decoction against chemotherapy-induced adverse effects [15]. Concurrently, the development of hydrogel-based delivery systems has opened new avenues for OA treatment. The integration of hydrogel technology with TCM self-assembled nanoparticles represents a promising approach to developing composite delivery systems that combine the advantages of targeted delivery, sustained release, and enhanced biological activity of herbal nanoparticles [16].

Vitamin A (retinol), an essential fat-soluble vitamin, regulates critical biological processes including cell proliferation and differentiation through active metabolites such as retinoic acid (RA) during vertebrate development [17]. Retinol metabolites promote the expression of extracellular matrix (ECM)-related genes in chondrocytes, suggesting a potential role for retinol metabolism in regulating cartilage homeostasis [18]. This provides evidence that retinol metabolism may contribute to the maintenance of articular cartilage integrity. Simultaneously, ferroptosis, a newly recognized form of regulated cell death characterized by iron-dependent lipid peroxidation, has been closely associated with OA progression [19]. Research demonstrates that chondrocyte ferroptosis serves as a significant driver of cartilage degeneration in OA [20].

This study successfully prepared and characterized self-assembled nanoparticles derived from T-SSAK (N-SSAK) and demonstrated their therapeutic efficacy, together with the hydrogel system loaded with N-SSAK (HA-MIX@N-SSAK), in the interleukin-1β (IL-1β) -stimulated chondrocyte model and destabilization of the medial meniscus (DMM)-induced mouse OA model. Furthermore, transcriptomic analysis was employed to investigate the underlying mechanisms of N-SSAK. Collectively, this work presents a novel intra-articular delivery platform based on self-assembled herbal nanoparticles, offering a promising therapeutic strategy for clinical OA management.

## 2 Results

### 2.1 Efficacy of T-SSAK in DMM-induced OA mice

**Fig. 1:**
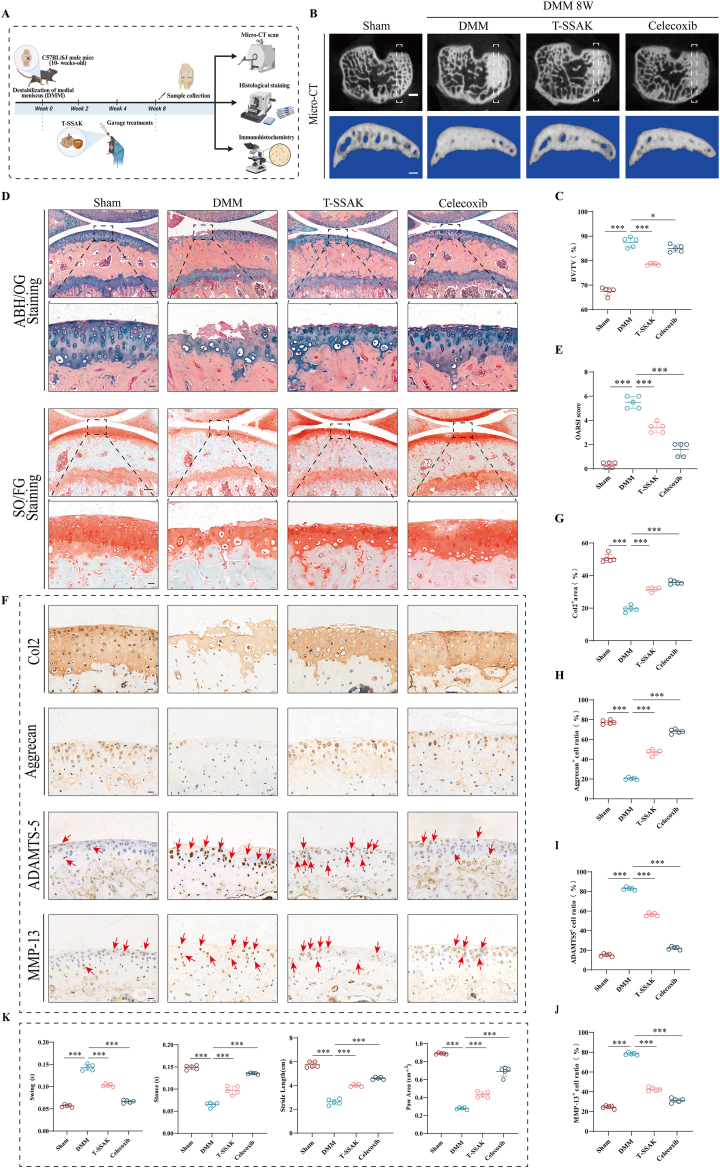
Efficacy of T-SSAK in DMM-induced OA mice. (A) Schematic diagram of animal experiments. (B) Micro-CT images of knee joints from different groups. n = 5. cale bar, 100 μm. (C) Quantitative analysis of the BV/TV of the subchondral bone. n = 5. (D) ABH/OG staining and SO/FG staining of knee joint sections from each group. n = 5. Scale bar, 20 μm. (E) OARSI score indicating the degree of knee joint degeneration in mice. (F) Representative IHC images of Col2, Aggrecan, ADAMTS-5, and MMP13 expression in knee cartilage. Red arrows indicate the specific sites of positive expression. (G-J) Quantification of Col2, Aggrecan, ADAMTS-5, and MMP13 positive cells in knee cartilage from different groups. n = 5. Scale bar, 20 μm. (K) Quantitative analysis of gait analysis for each group: swing time (s), stance time (s), stride length (cm), and paw area (cm^2^). n = 5. Values and error bars represent mean ± standard deviation. (ns: no significance, ∗: P < 0.05, ∗∗: P < 0.01, ∗∗∗: P < 0.001). (For interpretation of the references to color in this figure legend, the reader is referred to the Web version of this article.)

To evaluate the therapeutic effects of T-SSAK on OA, an OA mouse model was established using DMM surgery (Fig. 1A). Three-dimensional micro-CT reconstruction revealed that the T-SSAK and celecoxib administration significantly suppressed the aberrant subchondral bone remodeling in the tibial plateau of DMM model mice, such as elevating bone volume fraction (BV/TV) (Fig. 1B and C). Alcian blue hematoxylin/orange G (ABH/OG) staining and safranin O/fast green (SO/FG) staining showed that both T-SSAK and celecoxib significantly alleviated cartilage erosion, preserved cartilage thickness and matrix integrity, and reduced the osteoarthritis research society international (OARSI) score (Fig. 1D and E). IHC analysis further indicated that T-SSAK and celecoxib treatment upregulated the expression of cartilage matrix synthesis markers collagen-II (Col2) and Aggrecan, while downregulating the expression of cartilage matrix degradation markers a disintegrin and metalloproteinase with thrombospondin motifs 5 (ADAMTS-5) and matrix metalloproteinase 13 (MMP13) (Fig. 1F-J).

Joint pain responses were evaluated through pain-related behavioral and gait analyses (Fig. S1A). T-SSAK and celecoxib treatment significantly increased the mechanical paw withdrawal threshold (Fig. S1B), prolonged the thermal withdrawal latency (Fig. S1C), and restored the spontaneous locomotor activity of the mice (Fig. S1D). Moreover, gait analysis revealed that T-SSAK and celecoxib effectively ameliorated DMM-induced gait abnormalities, as evidenced by shortened swing time, prolonged stance time, and increased stride length and paw contact area (Fig. 1K). These findings suggest that T-SSAK alleviates OA-related joint damage and pain. Furthermore, liquid chromatography/mass spectrometry (LC/MS) analysis identified multiple bioactive components in the T-SSAK (Fig. S2A and B, Tables S1–2), and pathological staining confirmed its favorable biosafety profile (Fig. S3). Overall, T-SSAK showed similar effects on OA treatment as the commonly used drug celecoxib.

### 2.2 Preparation and characterization of self-assembled N-SSAK

**Fig. 2:**
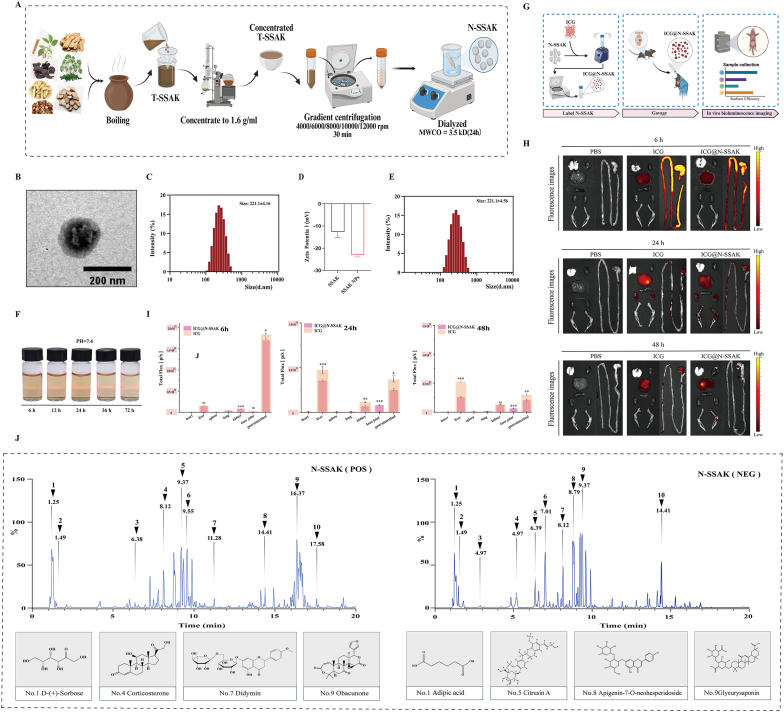
Preparation and characterization of self-assembled N-SSAK. (A) Schematic diagram of the preparation and characterization of self-assembled N-SSAK. (B) TEM image of N-SSAK. Scale bar, 200 nm. (C) Quantitative analysis of nanoparticle size. (D) Quantitative analysis of zeta potential. (E) Quantitative analysis of the size of lyophilized reconstituted nanoparticles. (F) Tyndall effect of N-SSAK in aqueous solutions at pH 7.4. (G) Schematic diagram of the N-SSAK *in vivo* imaging experiment. (H) Fluorescence images of heart, liver, spleen, lung, kidney, gastrointestinal tract, joint and bone tissues at predetermined time points (6, 24, and 48 h). n = 3. (I) Quantitative analysis of fluorescence intensity. (J) LC/MS analysis of N-SSAK in negative and positive mode and representative compounds. Values and error bars represent mean ± standard deviation. (ns: no significance, ∗: P < 0.05, ∗∗: P < 0.01, ∗∗∗: P < 0.001).

Given that self-assembled nanoparticles in TCM decoctions may serve as the pharmacological basis for their efficacy, we hypothesized that such nanostructures (designated N-SSAK) exist in T-SSAK and may be the key active components. According to the literature method [15], we successfully isolated T-SSAK nanoparticles through gradient centrifugation and dialysis (Fig. 2A). Transmission electron microscopy (TEM) revealed that N-SSAK exhibited a regular spherical morphology (Fig. 2B). The malvern particle size analyzer determined their nanoparticle size to be approximately 221.1 ± 4.16 nm (Fig. 2C), with a Zeta potential of −23.23 ± 2.14 mV (Fig. 2D). No significant change in particle size was observed after lyophilization and reconstitution, indicating good physical stability (Fig. 2E). To further evaluate the stability of N-SSAK under physiological conditions, we incubated the nanoparticles in buffers of different pH (1.2, 6.5, 7.4) for various durations (6-72 h) and monitored the Tyndall effect. The results showed no significant decrease in light scattering intensity under any condition, suggesting that the N-SSAK structure remains stable across a physiologically relevant pH range (Fig. 2F, Fig. S4A and B).

To investigate the distribution characteristics of N-SSAK *in vivo*, we tracked the distribution of ICG-labeled nanoparticles using an *in vivo* imaging system at 6, 24, and 48 h post-gavage. N-SSAK exhibited favorable oral stability, with fluorescence signals peaking at 24 h and persisting up to 48 h post-gavage. Notably, compared to free ICG, N-SSAK exhibited significantly enhanced accumulation in osteoarthritic bone, with distinct fluorescence observed in joint and bone tissues that peaked at 24 h post-injection, demonstrating its notable bone/joint tropism (Fig. 2G–I).

In addition, LC/MS analysis revealed that N-SSAK shared multiple bioactive components with the T-SSAK, such as liquiritin, didymin, adipic acid, indicating that N-SSAK effectively contained the primary active constituents of the decoction (Fig. 2J–Tables S3–4).

### 2.3 Molecular dynamics (MD) simulations of self-assembled N-SSAK in aqueous solution

**Fig. 3:**
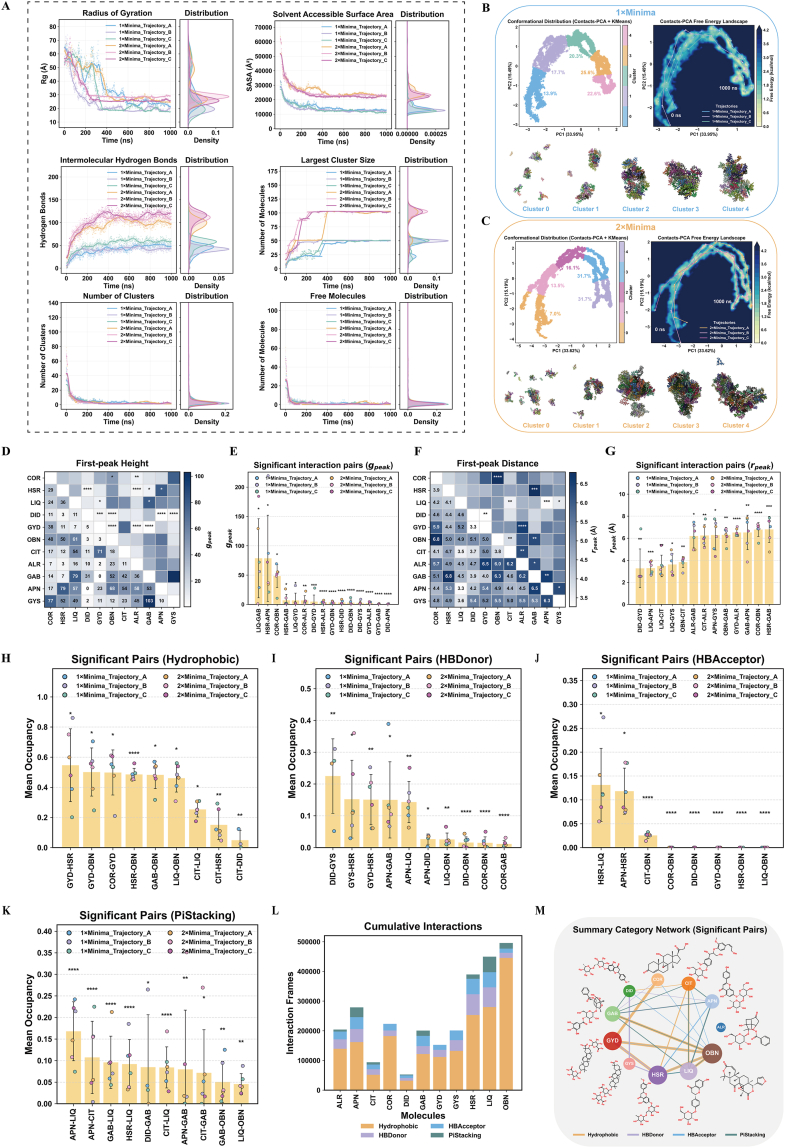
Molecular dynamics simulation of self-assembly for components in N-SSAK. (A) Quantitative characterization of “1x Minimal” and “2x Minimal” system, including radius of gyration, solvent accessible surface area, radius of gyration, and number of intermolecular hydrogen bonds, Largest Cluster Size, Number of Clusters, and Number of Free Molecules. (B) Structural evolution path of 1×Minimal self-assembly. (C) Structural evolution path of 2×Minimal self-assembly. (D) Heat map of the first peak height *g*_*peak*_ indexed by molecular species. (E) *g*_*peak*_ bar graph of “significant interaction pairs”. (F) Heat map of the first peak position *r*_*peak*_ (**Å**), indicating the most likely pairing spacing. (G) *r*_*peak*_ bar graph of “significant interaction pairs”. (H) Mean occupancy of significant pairs of hydrophobic. (I) Significant pairs of hydrogen bond donor. (J) Significant pairs of hydrogen bond acceptor. (K) Significant pairs of π-π stacking. (L) Cumulative Interactions of hydrophobic, hydrogen bond donor, hydrogen bond acceptor, and π-π stacking. (M)Summary Category Network. Values and error bars represent mean ± standard deviation. (ns: no significance, ∗: P < 0.05, ∗∗: P < 0.01, ∗∗∗: P < 0.001, ∗∗∗∗: P < 0.0001).

Through all-atom MD simulations, this study systematically elucidated the formation mechanism and structural basis of spontaneous self-assembled N-SSAK in aqueous solution. Analysis revealed that the radius of gyration and solvent-accessible surface area decreased significantly over time, indicating a transition from dispersed molecules to compact aggregates. Concurrently, the continuous increase in intermolecular hydrogen bonds reflected progressive maturation of the interaction network. The consistent increase in the largest cluster size, together with the simultaneous decrease in both the total cluster count and number of free molecules, provided clear evidence of an effective nucleation and growth process occurring within the system (Fig. 3A). Notably, the 2×Minimal system exhibited faster assembly kinetics but achieved a final structure essentially identical to the 1×Minimal system. Conformational space analysis uncovered the structural evolution pathway of self-assembly. Principal component analysis demonstrated that all trajectories evolved along the same contact-to-condensation axis in low-dimensional space. Free energy landscape analysis revealed highly overlapping minimum free energy regions under both concentration conditions, indicating that the self-assembly pathway and final structure were primarily governed by the intrinsic interaction landscape. Representative cluster conformations visually illustrated the structural transition from loose contacts (Cluster 0) to highly condensed states (Cluster 4) (Fig. 3B and C). Radial distribution function analysis identified several key molecular interaction pairs. The *g*_*peak*_ heatmap displayed local enrichment intensities for each molecular pair, with pairs such as LIQ-GAB and HSR-APN showing significant enrichment (Fig. 3D). The *r*_*peak*_ heatmap further indicated that these strong interaction pairs simultaneously exhibited short characteristic contact distances (3.3-4.5 Å) (Fig. 3E). Histogram analysis validated the robustness of these key molecular pairs across different concentration conditions, confirming their role as core structural units of the assembly (Fig. 3F and G). Further systematic analysis resolved the multimodal interaction network within the self-assembled system. Analysis of four distinct interaction categories, specifically hydrophobic, hydrogen bond donors, hydrogen bond acceptors, and π-π stacking, demonstrated that hydrophobic interactions constituted the fundamental structural framework of the assembly, whereas hydrogen bonds and π-π stacking contributed directional anchoring forces (Fig. 3H–K). Cumulative interaction bar charts identified OBN, LIQ, and HSR as the most active hub molecules participating in these interactions (Fig. 3L). Network diagrams comprehensively illustrated a stable topological architecture featuring a hydrophobic interaction framework reinforced by localized hydrogen bonding and π-π stacking points (Fig. 3M). Collectively, this study elucidated that the mechanism of self-assembled N-SSAK was driven by hydrophobic interactions as the backbone supplemented by selective π-π stacking and hydrogen bonding anchors. Hydrophobic interactions primarily mediated connectivity and condensation, while π-π stacking and hydrogen bonds provided orientation locking and localized reinforcement.

### 2.4 Efficacy of N-SSAK in DMM-induced OA mice

**Fig. 4:**
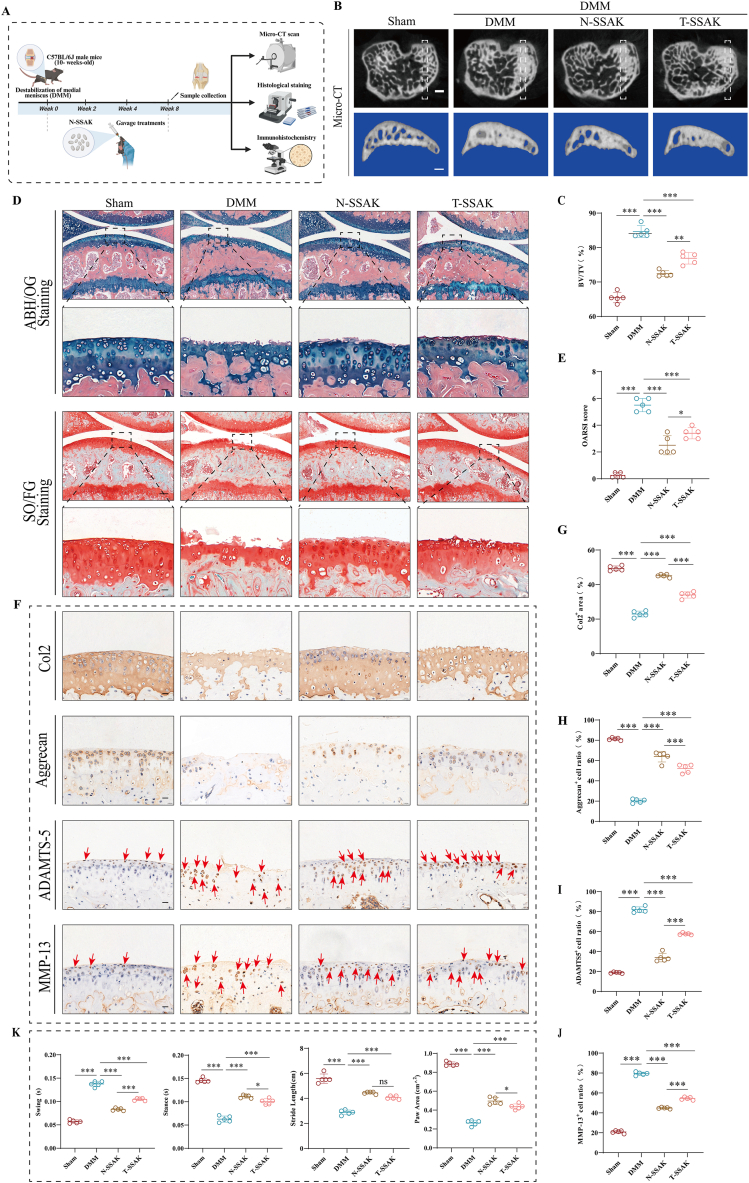
Efficacy of N-SSAK in DMM-induced OA mice. (A) Schematic diagram of animal experiments. (B) Micro-CT images of knee joints from different groups. n = 5. Scale bar, 100 μm. (C) Quantitative analysis of the BV/TV of the subchondral bone. n = 5. (D) ABH/OG staining and SO/FG staining of knee joint sections from each group. n = 5. Scale bar, 20 μm. (E) OARSI scores indicating the degree of knee joint degeneration in mice. (F) Representative IHC images of Col2, Aggrecan, ADAMTS-5, and MMP13 expression in knee cartilage. Red arrows indicate the specific sites of positive expression. (G-J) Quantification of Col2, Aggrecan, ADAMTS-5, and MMP13 positive cells in knee cartilage from different groups. n = 5. Scale bar, 20 μm. (K) Quantitative analysis of gait analysis for each group: swing time (s), stance time (s), stride length (cm), and paw area (cm^2^). n = 5. Values and error bars represent mean ± standard deviation. (ns: no significance, ∗: P < 0.05, ∗∗: P < 0.01, ∗∗∗: P < 0.001). (For interpretation of the references to color in this figure legend, the reader is referred to the Web version of this article.)

We next evaluated the ameliorative effect of N-SSAK on OA (Fig. 4A). Micro-CT scanning revealed that N-SSAK effectively suppressed abnormal subchondral bone remodeling and significantly reduced the BV/TV (Fig. 4B and C). Histochemical staining further confirmed that N-SSAK treatment effectively maintained cartilage structural integrity, improved matrix retention, and markedly decreased the OARSI score (Fig. 4D and E). In terms of cartilage metabolism, N-SSAK significantly upregulated the expression of Col2 and Aggrecan while downregulating ADAMTS-5 and MMP13, thereby contributing to the restoration of cartilage metabolic homeostasis (Fig. 4F–J). At the functional level, N-SSAK also showed notable efficacy in alleviating pain and improving motor function, as evidenced by significantly elevated mechanical withdrawal thresholds and thermal pain latency (Fig. S5A and B), as well as improved gait parameters (Fig. 4K). Furthermore, preliminary safety assessments confirmed the favorable biosafety profile of N-SSAK (Fig. S6). Collectively, these results indicate that N-SSAK significantly mitigate the pathological progression of OA in mice.

### 2.5 N-SSAK inhibit IL-1β-induced ECM damage in chondrocytes

**Fig. 5:**
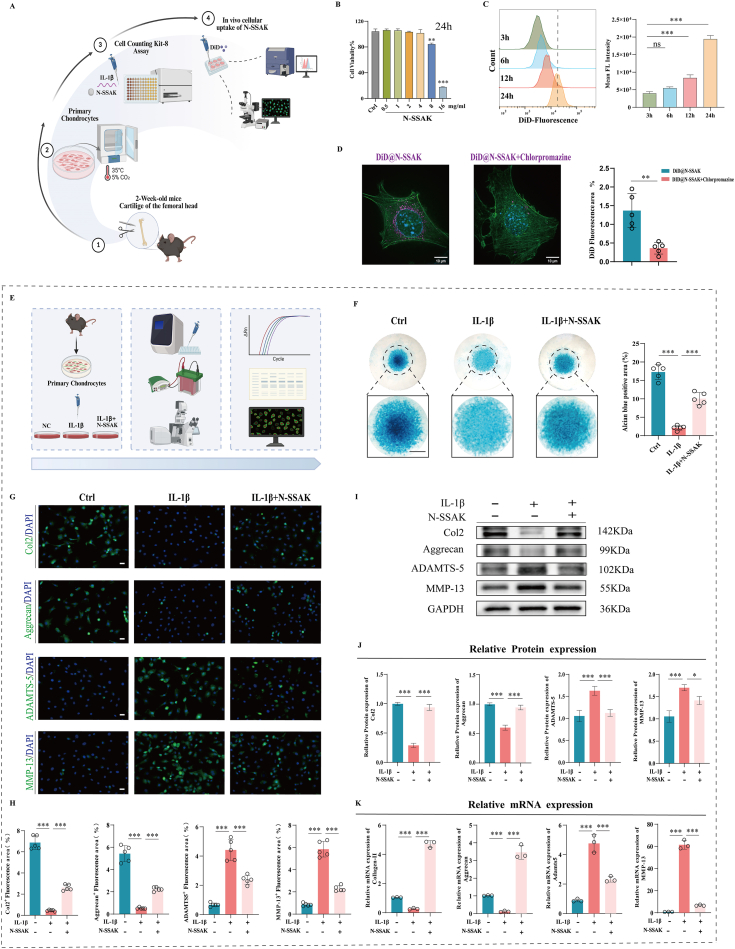
N-SSAK inhibit IL-1β-Induced ECM damage in chondrocytes. (A) Schematic diagram of the cellular uptake. (B) Cell activity was detected by CCK-8 assay at 24 h. (C) Flow cytometry analysis of cellular uptake of DID-labeled N-SSAK over 0-24 h and quantitative analysis of flow cytometry data. (D) Confocal microscopy image showing cellular uptake of DID-labeled N-SSAK in chondrocytes. (E) Schematic diagram of *in vitro* experiments. (F) Alcian blue staining for chondrocyte differentiation capacity. n = 5. Scale bar, 2 mm. (G) Representative IF images of Col2, Aggrecan, ADAMTS-5, and MMP13. n = 5. Scale bar, 20 μm. (H) Quantitative analysis of Col2, Aggrecan, ADAMTS-5, and MMP13 fluorescence area in each group. (I) WB bands of Col2, Aggrecan, ADAMTS-5, and MMP13 under different intervention conditions after IL-1β stimulation. n = 3. (J) Quantitative analysis of Col2, Aggrecan, ADAMTS-5, and MMP13 protein expression. (K) Quantitative RT-qPCR analysis of target gene expression. n = 3. Values and error bars represent mean ± standard deviation. (ns: no significance, ∗: P < 0.05, ∗∗: P < 0.01, ∗∗∗: P < 0.001). (For interpretation of the references to color in this figure legend, the reader is referred to the Web version of this article.)

We further investigated the protective effect of N-SSAK on OA chondrocytes *in vitro* (Fig. 5A). A safe and effective intervention concentration of 4 mg/mL N-SSAK was determined for subsequent experiments using the Cell counting Kit-8 (CCK-8) assay (Fig. 5B, Fig. S7A). Confocal microscopy and flow cytometry revealed efficient chondrocyte uptake of DiD-labeled N-SSAK, which was markedly suppressed by chlorpromazine, indicating that the uptake primarily relies on the clathrin-mediated endocytosis pathway (Fig. 5C and D, Fig. S7B). Alcian blue staining revealed that N-SSAK markedly promoted glycosaminoglycan synthesis (Fig. 5F). Consistent results from immunofluorescence (IF) staining, western blot (WB), and reverse transcription quantitative polymerase chain reaction (RT-qPCR) further confirmed that N-SSAK treatment significantly improved the ECM anabolic metabolism in IL-1β-stimulated chondrocytes (Fig. 5G–K). These findings collectively demonstrate that N-SSAK are taken up by chondrocytes via clathrin-mediated endocytosis, and inhibit the ECM degradation induced by IL-1β, thereby exerting remarkable chondroprotective effects.

### 2.6 N-SSAK Regulate Retinol Metabolism Pathway and Inhibit Chondrocyte Ferroptosis

**Fig. 6:**
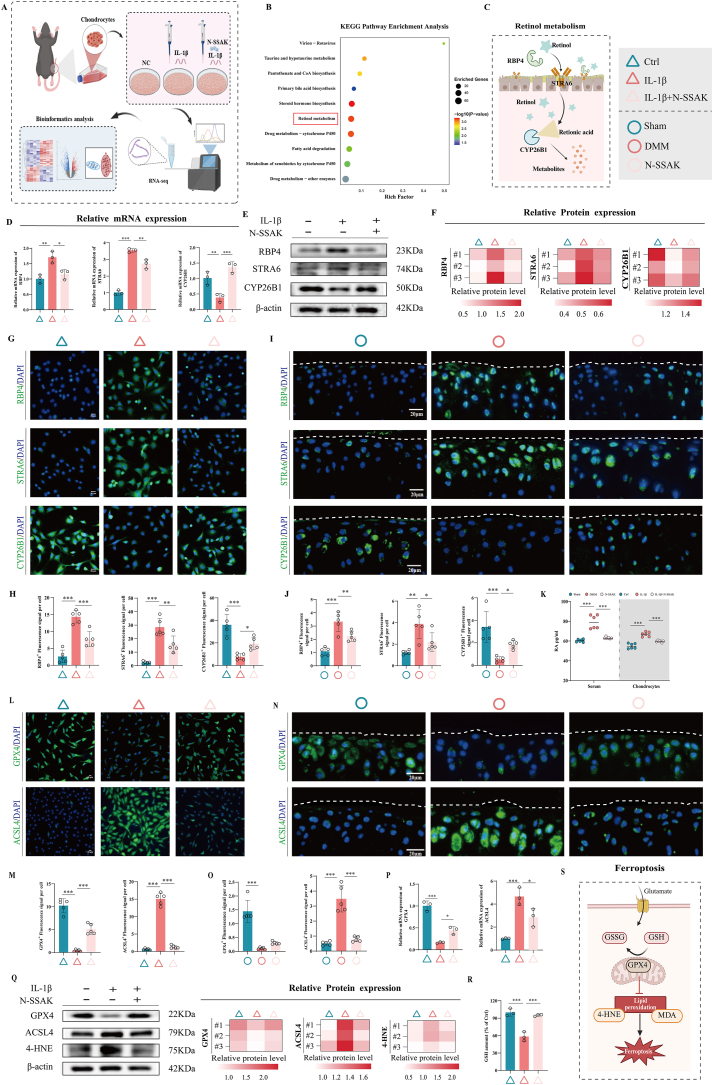
N-SSAK Regulate Retinol Metabolism Pathway and Inhibit Chondrocyte Ferroptosis. (A) Schematic diagram of *in vitro* experiments. (B) KEGG enrichment analysis of DEGs between the two groups. (C) Schematic diagram of retinol metabolism. (D) Quantitative RT-qPCR analysis of target gene expression. n = 3. (E) WB bands of RBP4, STRA6, and CYP26B1 under different intervention conditions after IL-1β stimulation. n = 3. (F) Quantitative analysis of RBP4, STRA6, and CYP26B1 protein expression. (G) Representative IF images of RBP4, STRA6, and CYP26B1 of chondrocytes. n = 5. Scale bar, 20 μm. (H) Quantitative analysis of RBP4, STRA6, and CYP26B1 fluorescence intensity in each group. (I) Representative IF images of RBP4, STRA6, and CYP26B1 of OA mice. n = 5. Scale bar, 20 μm. (J) Quantitative analysis of RBP4, STRA6, and CYP26B1 fluorescence intensity in each group. (K) RA expression in serum and chondrocytes was detected by ELISA. n = 6. (L) Representative IF images of GPX4 and ACSL4 of chondrocytes. n = 5. Scale bar, 20 μm. (M) Quantitative analysis of GPX4 and ACSL4 fluorescence intensity in each group. (N) Representative IF images of GPX4 and ACSL4 of OA mice. n = 5. Scale bar, 20 μm. (O) Quantitative analysis of GPX4 and ACSL4 fluorescence intensity in each group. (P) Quantitative RT-qPCR analysis of target gene expression. n = 3. (Q) WB analysis of GPX4, ACSL4, and 4-HNE proteins. n = 3. (R) Quantitative analysis of GSH level of each group. n = 3. (S) Schematic diagram of ferroptosis. Values and error bars represent mean ± standard deviation. (∗: P < 0.05, ∗∗: P < 0.01, ∗∗∗: P < 0.001).

To elucidate the mechanism of action of N-SSAK, we performed RNA sequencing analysis on chondrocytes stimulated with IL-1β for 24 h, comparing the gene expression profiles between the IL-1β group and the IL-1β + N-SSAK group (Fig. 6A). Using a threshold of p.adjust <0.05 and |log_2_FC| > 1, we identified a total of 523 differentially expressed genes, among which 271 genes were upregulated and 252 genes were downregulated (Fig. S8A and B). Principal component analysis demonstrated clear separation between the two experimental groups (Fig. S8C). Gene ontology (GO) enrichment analysis revealed significant enrichment of differentially expressed genes in RA catabolic processes (Fig. S8D), and Kyoto Encyclopedia of Genes and Genomes (KEGG) pathway analysis further indicated specific enrichment of the retinol metabolism pathway (Fig. 6B). Besides, GSEA indicated that compared to the IL-1β group, the ferroptosis pathway was significantly downregulated in the IL-1β + N-SSAK group (Fig. S8E).

Based on these findings, we focused on investigating the effects of N-SSAK on key proteins within the retinol metabolism pathway and ferroptosis. RT-qPCR and WB analysis demonstrated that IL-1β stimulation significantly upregulated RBP4 and STRA6 expression while suppressing CYP26B1 expression compared to the control group. N-SSAK intervention effectively reversed these alterations (Fig. 6D–F). CYP26A1 expression remained unaltered following N-SSAK treatment (Fig. S8F). IF staining further confirmed that N-SSAK significantly reduced IL-1β-induced RBP4 and STRA6 protein expression while promoting CYP26B1 expression (Fig. 6G and H). Similarly, *in vivo* experiments validated that DMM-induced OA mice exhibited elevated RBP4 and STRA6 expression as well as suppressed CYP26B1 expression in articular cartilage, all of which were significantly reversed by N-SSAK treatment (Fig. 6I and J). ELISA analysis revealed that the level of RA was markedly increased in the IL-1β-treated chondrocytes and in the serum of DMM-induced OA mice, while N-SSAK intervention significantly reduced these levels (Fig. 6K). These results suggest that N-SSAK suppress RA levels by inhibiting retinol metabolism.

To further demonstrate the effect of N-SSAK on ferroptosis, we assessed the markers related to ferroptosis. IF analysis showed that N-SSAK could reverse the downregulation of glutathione peroxidase 4 (GPX4) protein and the upregulation of acyl-CoA synthetase 4 (ACSL4) protein *in vivo* and *in vitro* (Fig. 6L–O). RT-qPCR and WB analysis revealed decreased GPX4 expression and increased ACSL4 levels following IL-1β stimulation, which were reversed by N-SSAK treatment (Fig. 6P and Q). Meanwhile, N-SSAK significantly reduced reactive oxygen species (ROS) accumulation both in IL-1β-stimulated chondrocytes and in articular cartilage of DMM-induced OA mice (Fig. S8G–J). In addition, we detected the lipid peroxidation products.Increased expression of 4-hydroxy-2-nonenal (4-HNE) and elevated levels of malondialdehyde (MDA) were observed in IL-1β-stimulated chondrocytes, and these increases were significantly attenuated by N-SSAK (Fig. 6Q, Fig. S8K). Additionally, glutathione (GSH) expression decreased in IL-1β-stimulated chondrocytes, but was markedly restored after N-SSAK treatment (Fig. 6R). To assess potential crosstalk between retinol metabolism and ferroptosis in N-SSAK action, we supplemented IL-1β-stimulated chondrocytes with exogenous RA. RA supplementation partially attenuated the N-SSAK-mediated restoration of COL2 expression in IL-1β-stimulated chondrocytes, whereas GPX4 and ACSL4 protein levels remained unaffected by RA (Fig. S9), indicating that the regulation of retinol metabolism and ferroptosis by N-SSAK may occur through largely independent pathways rather than through direct crosstalk.

These results systematically suggest that N-SSAK alleviate OA progression by modulating key molecules in the retinol metabolism pathway, restoring RA levels, and inhibiting chondrocyte ferroptosis.

### 2.7 Preparation and characterization of the hydrogel-based drug delivery system HA-MIX@N-SSAK

**Fig. 7:**
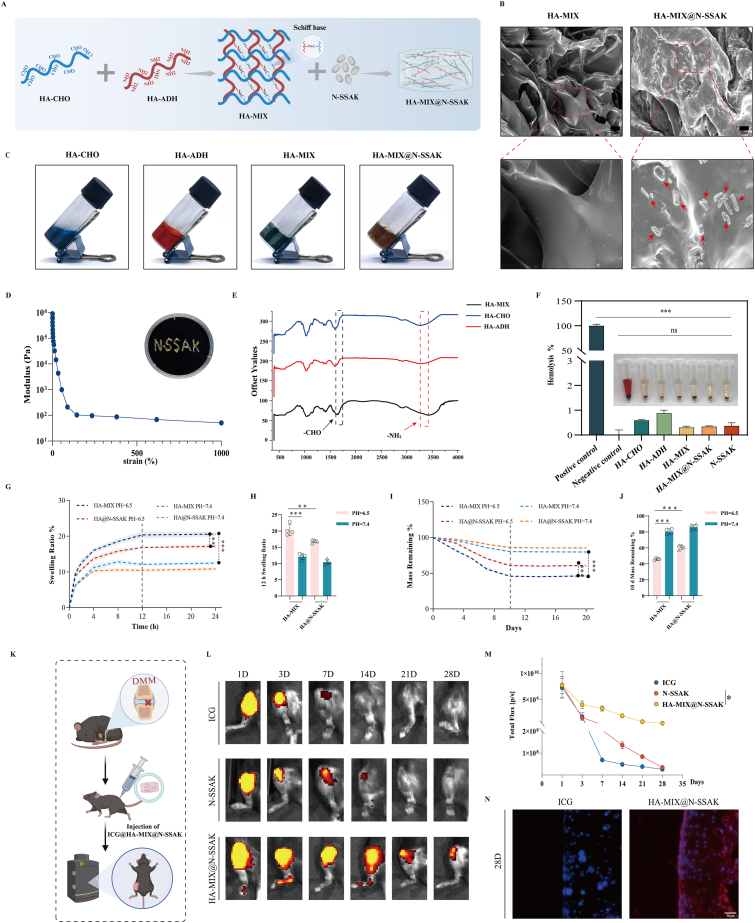
Preparation and characterization of the hydrogel-based drug delivery system HA-MIX@N-SSAK. (A) Schematic diagram of the preparation of the hydrogel-based drug delivery system HA-MIX@N-SSAK. (B) Scanning electron micrographs of HA-MIX and HA-MIX@N-SSAK. Red arrows indicate N-SSAK. (C) Digital photos showing the formation of HA-MIX by mixing HA-CHO and HA-ADH solution. (D) Demonstration of the injectability and Young's modulus of HA-MIX@N-SSAK. (E) FTIR spectra of the HA-CHO, HA-ADH, and HA-MIX. (F) Hemolysis rate and photographs of hemolysis of the HA-CHO, HA-ADH, HA-MIX, HA-MIX@N-SSAK, and N-SSAK. (G) The swelling behavior of the HA-MIX and HA-MIX@N-SSAK under different pH conditions. n = 4. (H) Quantitative analysis of the swelling behavior of the HA-MIX and HA-MIX@N-SSAK under different pH at 12 h. (I) The degradation behavior of the HA-MIX and HA-MIX@N-SSAK under different pH conditions. n = 4. (J) Quantitative analysis of the degradation behavior of the HA-MIX and HA-MIX@N-SSAK under different pH conditions at 10 d. (K) Schematic diagram of the ICG@HA-MIX@N-SSAK *in vivo* imaging experiment. (L) *In vivo* fluorescence imaging of the knee of mice. (M) Quantitative analysis of *in vivo* fluorescence intensity. (N) Fluorescence images of knee cryosections displaying the distribution of ICG@HA-MIX@N-SSAK 28 d post-injection. The nuclei were stained with DAPI. n = 3, Scale bar, 50 μm. Values and error bars represent mean ± standard deviation. (ns: no significance, ∗: P < 0.05, ∗∗: P < 0.01, ∗∗∗: P < 0.001). (For interpretation of the references to color in this figure legend, the reader is referred to the Web version of this article.)

Based on the better effect of N-SSAK in OA treatment, we successfully developed an injectable hydrogel-based drug delivery system, designated HA-MIX@N-SSAK, for intra-articular administration (Fig. 7A). Scanning electron microscopy (SEM) revealed that N-SSAK were uniformly distributed within the HA-MIX@N-SSAK hydrogel and maintained their characteristic morphology, whereas no such nanostructures were observed in the HA-MIX group (Fig. 7B, Fig. S10A). The hydrogel formed to HA-MIX rapidly at room temperature via Schiff base reaction between the aldehyde groups of aldehyde-modified hyaluronic acid (HA-CHO) and the amino groups of amine-modified hyaluronic acid (HA-ADH) (Fig. 7C). The resulting hydrogel exhibited excellent injectability through a 26-gauge needle and rapidly recovered its structural integrity post-injection (Fig. 7D). Simultaneously​, as the shear strain increased, the HA-MIX@N-SSAK gel showed shear thinning behavior, which supported its suitability for intra-articular injection via syringe (Fig. 7D) [21]. Additionally, the HA-MIX@N-SSAK gel possessed both rapid self-healing ability after shear dilution and strong tissue adhesion capability, allowing it to firmly adhere to organs and bone (Fig. S10B and C). FTIR spectroscopy further confirmed the successful synthesis of both HA-CHO and HA-ADH (Fig. 7E).

Hemocompatibility assessment showed clear supernatants and hemolysis rates below 5% for HA-CHO, HA-ADH, N-SSAK, and HA-MIX@N-SSAK groups, indicating favorable blood compatibility (Fig. 7F) [22]. Under simulated OA conditions of weak acidity, the Schiff base linker easily breaks down, and the swelling and degradation of the hydrogel are key factors affecting micelle or drug release [23]. *In vitro* swelling tests demonstrated significantly enhanced swelling ratio under OA-mimetic acidic environment (pH 6.5) [24] compared to physiological environment (pH 7.4) (Fig. 7G and H). Degradation studies revealed a substantially lower mass retention rate (45.925%) at pH 6.5 after 10 days versus that at pH 7.4 (80.45%), with HA-MIX@N-SSAK showing an appropriately moderated degradation pattern (Fig. 7I and J).

*In vivo* imaging results demonstrated that the hydrogel significantly prolonged the retention of N-SSAK in the joint cavity, with strong DiD fluorescence signals still detectable on day 28 post-injection (Fig. 7L and M). Cryosectioning of joint tissues further confirmed substantial presence of DiD-labeled N-SSAK within the cartilage matrix at day 28, indicating effective release of the nanoparticles from the hydrogel (Fig. 7N).

In summary, HA-MIX@N-SSAK exhibited excellent injectability, biocompatibility, pH-responsive degradation, and sustained release capabilities, representing a promising intra-articular drug delivery system for OA treatment.

### 2.8 Therapeutic effects of HA-MIX@N-SSAK in DMM-induced OA mice

**Fig. 8:**
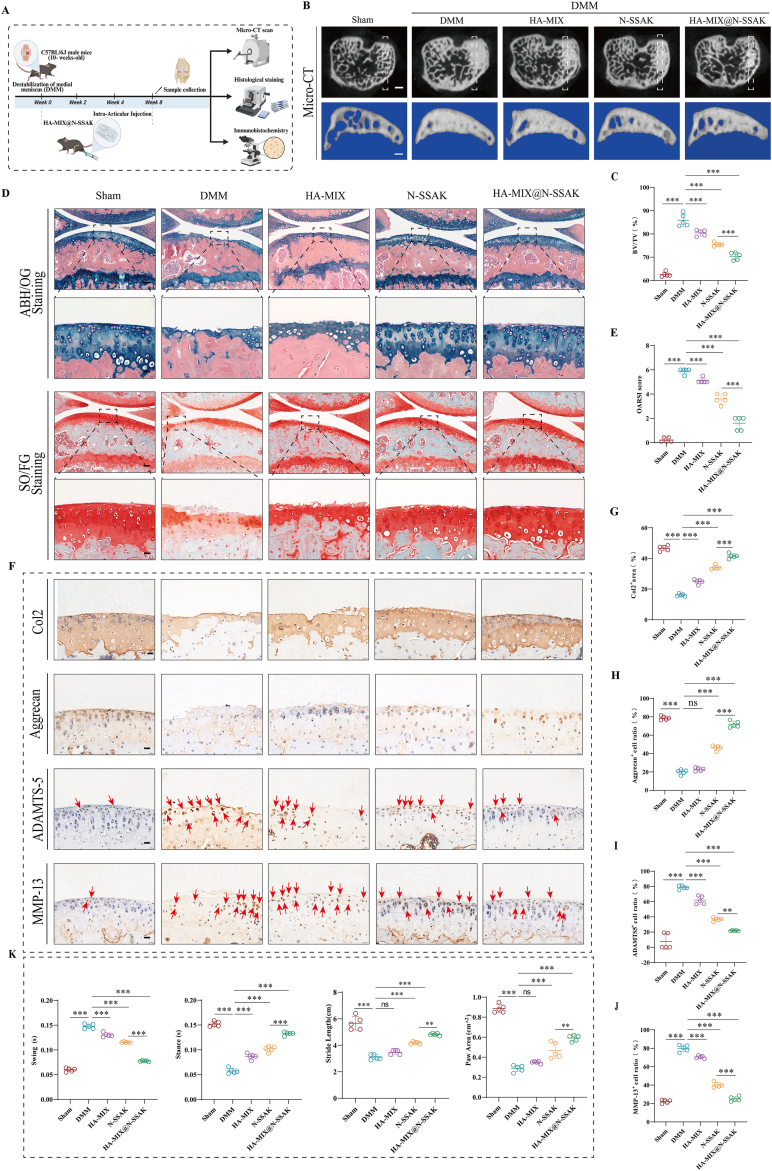
Therapeutic effects of HA-MIX@N-SSAK in DMM-induced OA mice. (A) Schematic diagram of animal experiments. (B) Micro-CT images of knee joints from different groups. n = 5. Scale bar, 100 μm. (C) Quantitative analysis of the BV/TV of the subchondral bone. n = 5. (D) ABH/OG staining and SO/FG staining of knee joint sections from each group. n = 5. Scale bar, 20 μm. (E) OARSI score indicating the degree of knee joint degeneration in mice. (F) Representative IHC images of Col2, Aggrecan, ADAMTS-5, and MMP13 expression in knee cartilage. Red arrows indicate the specific sites of positive expression. (G-J) Quantification of Col2, Aggrecan, ADAMTS-5, and MMP13 positive cells in knee cartilage from different groups. n = 5. Scale bar, 20 μm. (K) Quantitative analysis of gait analysis for each group: swing time (s), stance time (s), stride length (cm), and paw area (cm^2^). n = 5. Values and error bars represent mean ± standard deviation. (ns: no significance, ∗: P < 0.05, ∗∗: P < 0.01, ∗∗∗: P < 0.001). (For interpretation of the references to color in this figure legend, the reader is referred to the Web version of this article.)

To further enhance the therapeutic effect, we developed a hydrogel-based Chinese herbal formula nanoparticle system (HA-MIX@N-SSAK) and evaluated its efficacy in treating OA (Fig. 8A). Three-dimensional micro-CT reconstruction demonstrated that HA-MIX@N-SSAK significantly ameliorated abnormal subchondral bone remodeling in the tibial plateau, with effects comparable to the sham group and superior to single treatments with HA-MIX or N-SSAK in reducing BV/TV (Fig. 8B and C). Histological analysis revealed that the HA-MIX@N-SSAK group achieved the most preserved cartilage structural integrity with significantly reduced OARSI score, whereas HA-MIX or N-SSAK monotherapy provided only partial improvement (Fig. 8D and E). IHC results indicated that HA-MIX@N-SSAK markedly upregulated the expression of Col2 and Aggrecan, while suppressing ADAMTS-5 and MMP13. Notably, HA-MIX alone failed to effectively elevate Aggrecan protein levels (Fig. 8F–J).

In behavioral tests, HA-MIX@N-SSAK significantly increased the mechanical paw withdrawal threshold (Fig. S11A) and thermal pain latency (Fig. S11B). Additionally, it effectively normalized gait parameters, including reduced swing time, prolonged stance time, and increased stride length and paw print area (Fig. 8K). In contrast, HA-MIX alone showed limited effectiveness in gait improvement. Besides, pathological examination confirmed the favorable biosafety profile of HA-MIX@N-SSAK (Fig. S12). These results showed that HA-MIX@N-SSAK synergistically alleviated structural damage and pain responses in OA, with efficacy superior to that of individual component treatments.

## 3 Discussion

OA represents a common degenerative joint disorder that substantially compromises patients’ daily functioning and imposes socioeconomic and healthcare burdens. Articular cartilage degradation constitutes a core pathophysiological hallmark of disease progression, highlighting the clinical imperative for disease-modifying interventions that can arrest and reverse this degeneration [25]. Our study established a progressive research framework encompassing traditional decoction (T-SSAK), self-assembled nanoparticles (N-SSAK), and hydrogel-based composite delivery systems (HA-MIX@N-SSAK). The progression from T-SSAK to N-SSAK and finally to HA-MIX@N-SSAK follows a deliberate stepwise optimization strategy. T-SSAK serves as the clinical foundation, an empirically effective herbal formula whose therapeutic potential is limited by poor bioavailability and inadequate targeting capability. Isolating N-SSAK from this decoction transforms the multi-component herbal mixture into a stable, carrier-free nanoparticle system, which enhances solubility, prolongs circulation, and enriches the pharmacologically active constituents. Incorporating N-SSAK into the HA-MIX hydrogel then enables sustained local release and pH-responsive drug delivery within the inflammatory osteoarthritic microenvironment. Furthermore, our Mechanistic investigations revealed that N-SSAK alleviated OA progression by modulating the retinol metabolism pathway and inhibiting chondrocyte ferroptosis. This study provides a novel multi-target therapeutic strategy rooted in TCM principles for nanomaterial-based OA intervention.

Modern pharmacological research has demonstrated that multiple active components in SSAK decoction possess antioxidant, anti-inflammatory, inhibiting chondrocyte ferroptosis, and anti-cartilage degeneration properties. For instance, didymin, a natural flavonoid glycoside, has been identified as a novel regulator of ferroptosis in knee OA [26]. Active compounds from eucommia bark protect chondrocytes from IL-1β-induced damage through antioxidant effects that reduce oxidative stress and modulating pro-inflammatory cytokine signaling pathways [27]. Components from two-toothed achyranthes root regulate multiple signaling pathways, including TNF, PI3K/AKT, and IL-17, thereby exerting anti-inflammatory effects against OA [28]. Astragaloside IV suppresses chondrocyte ferroptosis via the NRF2 pathway, while constituents from rehmannia root and Chinese angelica inhibit extracellular matrix degradation and oxidative stress through MAPK signaling, consequently ameliorating cartilage degeneration [29]. Our study confirmed that T-SSAK significantly delayed cartilage damage progression, as evidenced by reduced OARSI score, upregulated COL2 and Aggrecan expression, and downregulated MMP-13 and ADAMTS-5 expression. Imbalance in cartilage matrix metabolism represents a core aspect of OA pathogenesis [30]. Under physiological conditions, chondrocytes maintain matrix homeostasis through coordinated anabolic and catabolic processes. However, this equilibrium is disrupted in OA, leading to increased catabolic enzyme expression and decreased anabolic protein production [31]. Our data showed that T-SSAK restored metabolic balance and promote cartilage regeneration. Additionally, abnormal subchondral bone remodeling constitutes another crucial pathological feature of OA, closely associated with cartilage degeneration [32]. Articular cartilage and subchondral bone function as an integrated functional unit that collectively participates in disease progression [33]. Micro-CT analysis in our study demonstrated that T-SSAK ameliorated abnormal subchondral bone remodeling in OA mice, suggesting that it indirectly facilitates cartilage repair and protection by optimizing bone microstructure. The subchondral bone region, rich in blood vessels and nerves, serves as an important source of OA-related pain [34]. As a primary clinical manifestation of OA, pain represents the main reason for patients seeking medical attention. Our behavioral and gait analyses showed that T-SSAK could effectively alleviate pain sensitivity and improve gait abnormalities in OA mice. The mechanisms through which T-SSAK improves abnormal subchondral bone remodeling and alleviates pain in OA will be the focus of our subsequent research investigations.

While preliminary research and clinical practice have confirmed the favorable therapeutic efficacy of T-SSAK against OA, traditional decoctions still face application challenges such as low bioavailability, poor targeting efficiency, and unsatisfactory palatability [35]. Exploiting the innate self-assembly of herbal constituents to form nanoparticles enhances component synergy, yielding improved bioavailability and therapeutic efficacy [36]. Recent studies have shown that the co-boiling of Astragalus and Angelica can form self-assembled nanoparticles, exhibiting anti-cardiac fibrosis activity [37]. Similarly, the decoction of licorice root has been shown to self-assemble into nanoparticles that could promote the proliferation of normal hepatocytes [38]. However, current researches predominantly focus on two-herb interactions, with limited exploration of self-assembly in multi-herb formulations. In this study, we successfully isolated self-assembled nanoparticles (N-SSAK) from T-SSAK using differential centrifugation. Unlike conventional herbal preparations, the nanoparticles in nanostructured self-assemblies are derived entirely from natural bioactive molecules of traditional Chinese medicine. Therefore, they exhibit excellent biocompatibility and innate affinity for biological systems [39]. Furthermore, the self-assembly process avoids toxic solvent residues and complex purification steps [13]. To note, the stability of nanoparticles is a key determinant of their overall performance [40]. N-SSAK exhibited consistent and excellent dispersibility and structural integrity under various pH conditions and over time. Meanwhile, MD results showed that hydrophobic interactions, hydrogen bonding, and π-π stacking served as the primary driving forces for the formation of N-SSAK. Specifically, within N-SSAK, obacunone (OBN), liquiritin (LIQ), and hesperetin 7-O-rutinoside (HSR) served as hub molecules characterized by high connectivity and substantial proportional representation. These hub molecules are not merely structural scaffolds but are also documented pharmacologically active agents against OA. For example, OBN alleviates OA progression by inhibiting p38MAPK signaling [41]. LIQ protects chondrocytes from apoptosis via P53/PUMA pathway activation [42]. HSR exerts anti-inflammatory effects on IL-1β-treated human chondrocytes [43]. Moreover, the identification of OBN, LIQ and HSR as hub molecules aligned well with our subsequent mechanistic data, as these compounds were reported to suppress ferroptosis, thereby providing a plausible molecular link between the nanostructure composition and the observed inhibition of chondrocyte ferroptosis [[44], [45], [46]]. These key constituents simultaneously engaged in hydrophobic interactions, hydrogen bonding, and π-π stacking, thereby establishing the fundamental network architecture and facilitating intermodal connections. Meanwhile, apigenin-7-O-neohesperidosid (APN), citrusin A (CIT), 4-O-Galloylalbiflorin (GAB), and didymin (DID) acted as selective molecules that provided directional reinforcement within π-π or hydrogen bonding channels. These findings systematically elucidate the self-assembly nanonization of a complex formula comprising over ten herbal components, thereby better reflecting the combinatorial principle of the “Jun-Chen-Zuo-Shi” in TCM. Furthermore, through *in vitro* and *in vivo* experiments, we validated the significant protective effects of N-SSAK on chondrocytes and the OA pathological process.

Retinol metabolism plays a crucial role in maintaining cellular differentiation, proliferation, and homeostasis [47]. Through transcriptomic analysis, our study identified the retinol metabolism pathway as a central mechanism by which N-SSAK exerted its therapeutic effects. As a lipophilic micronutrient, retinol can be converted into various bioactive metabolites that participate in physiological regulation through gene expression modulation [48]. Clinical studies have suggested that the concentration of retinol was positively correlated with the risk of OA and excessive retinol might be a causative factor for OA [49]. Retinol is stored in an esterified form and specifically transported to target cells via RBP4 [50]. Cellular uptake is then mediated by the retinol signaling receptor STRA6, followed by its conversion into bioactive form, RA. Research has indicated that both RBP4 and STRA6 were expressed in the joint tissues of patients with OA, with their levels correlating with the increase of matrix degrading enzymes in chondrocytes [51]. RA can increase the expression of MMP13 and reduce the expression of COL2A1, thereby regulating chondrocyte catabolism [52]. In the OA pathological environment, we also observed significant suppressed expression of the metabolic enzyme CYP26B1, leading to the excessive accumulation of RA and consequent disruption of chondrocyte metabolic homeostasis. Importantly, N-SSAK showed bidirectional regulatory capacity by simultaneously inhibiting abnormal overexpression of RBP4/STRA6 as well as increasing the expression of CYP26B1, thereby restoring physiological RA levels and reestablishing metabolic balance. Besides, the selective regulation of CYP26B1 rather than CYP26A1 by N-SSAK may be related to the distinct tissue distribution of these RA-metabolizing enzymes and the genetic association of CYP26B1 with OA susceptibility [53,54]. Furthermore, GSEA analysis revealed that ferroptosis is another important mechanism of action for N-SSAK. Ferroptosis, an iron-dependent form of programmed cell death, plays a critical role in OA cartilage degeneration [55]. GSH prevents the chain reaction of lipid peroxidation via GPX4, and a decrease in GSH levels or inhibition of GPX4 activity worsens lipid peroxidation [56]. In ferroptosis, lipid peroxidation primarily takes place within the lipid bilayers of cellular membranes, compromising their structural integrity and generating toxic secondary products including 4-HNE and MDA [57]. Studies suggested that IL-1β stimulation induced ferroptosis in chondrocytes [58,59]. Our data support that IL-1β activated ferroptosis, while N-SSAK effectively inhibited lipid peroxidation and reduced ferroptosis in chondrocytes. Overall, these results indicate that N-SSAK exerts chondroprotective effects through dual inhibition of retinol metabolism and ferroptosis.

Despite the considerable promise of self-assembled nanotherapeutics derived from natural herbs, their clinical translation is hindered by carrier dependency and insufficient intrinsic targeting capabilities [60]. To address these limitations, we further developed an injectable hydrogel-based drug delivery system, HA-MIX@N-SSAK, for intra-articular administration to enhance clinical therapeutic outcomes. Existing evidence indicates that direct intra-articular injection of nanomedicines is constrained by short retention time, which limits long-term efficacy [61]. In contrast, as three-dimensional hydrophilic networks capable of substantial water absorption and retention, hydrogels not only achieve effective encapsulation of nanomaterials but also enable controlled release of active components through spontaneous or triggered mechanisms, thereby offering superior treatment options for OA [62,63]. In this study, intra-articular administration of HA-MIX@N-SSAK in OA mice achieved sustained N-SSAK release for up to 28 days. Compared to oral administration, this delivery system significantly improved drug targeting and local concentration, effectively suppressing the progression of cartilage degeneration in OA. Evidence from prior research indicates that intra-articular injection is superior to oral administration for drug delivery [64]. Notably, the Schiff base bonds within HA-MIX@N-SSAK exhibited pH-responsive characteristics, enabling rapid drug release in the inflammatory microenvironment of OA for early intervention. Supporting evidence from Liu et al. [23]. validated accelerated release from hydrogels at pH 6.8, confirming their potential as pH-responsive carriers. Furthermore, the physicochemical properties of the hydrogel closely resemble those of synovial fluid, potentially providing sustained lubrication and mechanical support for damaged cartilage [65]. Therefore, the HA-MIX@N-SSAK hydrogel system developed in this study shows significant promise for clinical translation, owing to its sustained-release profile, enhanced targeting efficacy, and pathologically-responsive drug release behavior.

Despite the valuable findings of this study, several limitations warrant attention. First, although the surgical DMM-induced OA mouse model replicates numerous pathological features of human OA, the acute injury mechanism may differ from the chronic natural progression of the human disease. Second, the exclusive use of young male mice precludes evaluation of how age and sex factors might influence therapeutic outcomes. Future studies should aim to increase sample diversity to validate the generalizability of the results. Additionally, the *in vivo* metabolic pathways and long-term biocompatibility of N-SSAK require further exploration and assessment. Moreover, the MD simulation input ratios derived from LC/MS data may not fully represent the actual molecular packing or functional contribution of each compound within the assembled nanoparticles. Finally, future siRNA-based validation of molecules such as RBP4 will be helpful to establish their causal roles in mediating the dual effects of N-SSAK.

## 4 Conclusion

In conclusion, we demonstrated the protective effect of self-assembled nanoparticles derived from active components of the SSAK decoction on OA. N-SSAK could regulate retinol metabolism pathways and inhibit chondrocyte ferroptosis, thereby restoring cartilage metabolic homeostasis, suppressing abnormal subchondral bone remodeling, and alleviating pain and functional impairment. To achieve a better therapeutic effect, we further developed a hydrogel-based system (HA-MIX@N-SSAK) that enabled targeted intra-articular delivery and sustained release of N-SSAK nanoparticles. This study provides a novel TCM-derived nanotherapeutic strategy for OA intervention and offers compelling evidence supporting the clinical translation of self-assembled nanoparticles from natural herbs.

## 5 Materials and methods

### 5.1 Materials

The SSAK decoction consisted of 10 natural herbs, which were purchased from Zhejiang Chinese Medical University Traditional Chinese medicine pieces Co., Ltd. (Zhejiang, China). Celecoxib (8145742) was purchased from Pfizer (USA). Hyaluronate sodium (HA; MW = 800-1500 kDa, H909938), N, N-Dimethylformamide (DMF; N807507), sodium periodate (NaIO4; S817518), ethylene glycol (E808735), adipic acid dihydrazide (ADH; A800779), N-(3-Dimethylaminopropyl)-N′-ethylcarbodiimide hydrochloride (EDC; N808856), 1-Hydroxybenzotriazole (HBOT; H742557), and D-Mannitol (M6266) were purchased from Macklin (China). IL-1β (211-11B) was purchased from PeproTech. COL2 (ab34712), MMP-13 (ab39012) and 4-HNE (ab39012) were purchased from Abcam. Aggrecan (bs-11655R) and ADAMTS-5 (bs-3573R) were purchased from Bioss. GPX4 (A13309) and ACSL4 (A20414) were purchased from Abclonal. RBP4 (84503-4-RR), STRA6 (22001-AP), and CYP26B1 (21555-1-AP) were purchased from Proteintech. Alexa Fluor® 488 labeled goat anti-rabbit IgG (ab150077) was obtained from Abcam. DAPI (C1006), CCK-8 detection kit (C0040), GSH and GSSG assay kit (S0053), ROS assay kit (S0033S), lipid peroxidation MDA assay kit (S0131S), and DiD (C1039) were purchased from Beyotime. RA (HY-14649) was purchased from MCE. RA detection kit (JRXW206196) was obtained from Ruixin Biotechnology Co., Ltd. (Fujian, China). Indocyanine green (ICG; R018175) was purchased from Yien Chemical Technology Co., Ltd. (Shanghai, China). Chlorpromazine (R004754-5g) was purchased from RHAWN.

### 5.2 Preparation of T-SSAK

T-SSAK (Eucommia bark 10g, rehmannia root 15g, astragalus root 20g, Chinese angelica 12g, two-toothed achyranthes root 12g. chuanxiong root 9g, cornus 12g, bitter orange 10g, white peony root 15g, and licorice root 5g) was prepared through reflux extraction, followed by filtration and concentration to obtain a stock solution with a crude drug concentration of 1.6 g mL^−1^.

### 5.3 Preparation and characterization of N-SSAK

N-SSAK was prepared and characterized following previously established methods [15]. The T-SSAK was subjected to sequential gradient centrifugation at 4 °C (4000, 6000, 8000, 10000, and 12000 rpm, 30 min each). The supernatants were pooled and subsequently centrifuged again to remove precipitates. The resulting supernatant was dialyzed against deionized water using a 3.5 kDa molecular weight cutoff dialysis membrane (YA1054, Solarbio), followed by filtration through a 0.22 μm filter. The final product, designated as N-SSAK, was lyophilized and stored under dry conditions at 4 °C for subsequent experiments.

The ultrastructure of N-SSAK was characterized using cryogenic transmission electron microscopy (Cryo-TEM; Tecnai G2 F20, USA). The hydrodynamic diameter distribution and zeta potential were determined using a Malvern Nano Zeta potential and particle size analyzer (Zetasizer Nano ZSE, UK).

### 5.4 MD simulation of the formation mechanism of N-SSAK in aqueous solution

This study employed all-atom molecular dynamics simulations to investigate the formation mechanism of N-SSAK in aqueous solution. Based on the relative concentrations of 11 natural compounds identified in N-SSAK (Table S5) through LC/MS analysis, we established two initial concentration systems designated as 1×Minimal (minimum baseline concentration) and 2×Minimal. Initial configurations were generated using Packmol with minimum intermolecular distances ≥8.0 Å. Molecular parameterization was performed in Amber24 using the GAFF2 force field, with ABCG2 atomic charges calculated via the AM1-BCC method. Systems were explicitly solvated in the TIP3P water model with appropriate counterions. The simulation protocol comprised two-stage energy minimization, NVT equilibration to 300 K, 1 ns NPT equilibration, and 1000 ns production simulation. Hydrogen mass repartitioning was employed during the production phase to enable a 4 fs timestep. Trajectory analysis was performed using CPPTRAJ. Conformational evolution was characterized through principal component analysis and free energy landscape construction. Significant molecular pairs and their contact characteristics were identified using radial distribution functions, while ProLIF was utilized to quantify occupancy rates of hydrophobic interactions, hydrogen bonds, and π-π stacking, enabling the construction of molecular interaction networks. All analyses were based on three independent replicate trajectories for each concentration condition. Statistical tests were FDR-corrected to ensure the robustness of the results [[66], [67], [68]].

### 5.5 Preparation of modified HA

800 mg of HA was dissolved in 90 mL of deionized water. After complete dissolution, 450 mg of NaIO4 was added to the solution, and the reaction proceeded for 4 h. Subsequently, the reaction was quenched by adding 0.5 mL of ethylene glycol, yielding HA-CHO. For HA-ADH, 2000 mg of HA was dissolved in deionized water, followed by the addition of 4580 mg of ADH. Subsequently, 2020 mg of EDC and 1420 mg of HOBT were added dropwise sequentially. The pH of the reaction mixture was then adjusted to 6.8 using 2 M hydrochloric acid, and the reaction was allowed to proceed overnight. Next, the synthesized HA-CHO and HA-ADH were mixed at a specific ratio and stirred at room temperature until a homogeneous hydrogel formed, yielding the final HA-MIX product.

### 5.6 Synthesis and characterization of cross-linked HA loaded with Chinese herbal formula nanoparticles

A composite hydrogel, designated HA-MIX@N-SSAK, was prepared by dissolving 600 mg of HA-CHO and 6200 mg of HA-ADH in 1 mL of a resuspension solution containing the Chinese herbal formula nanoparticles, followed by stirring at 500 rpm for 5 min.

The chemical structure of the hydrogel was analyzed using FTIR spectroscopy (Nicolet iS50, Thermo Fisher Scientific, USA). The microscopic structure of the hydrogel was analyzed using a scanning electron microscope (SEM; Hitachi, Japan). A hydrogel specimen measuring 0.1 cm × 0.4 cm × 0.1 cm was mounted on a sample stub and rapidly cryo-immobilized by immersion in liquid nitrogen to preserve its native hydrated architecture. Following cryofixation, the sample was transferred to a cryo-preparation chamber where it was fractured to reveal internal structure. Surface ice was removed by sublimation in a freeze-dryer for 3 min, followed by dual-layer gold sputter coating (10 mA, 45 s per cycle). Surface morphology examination was subsequently conducted on the low-temperature stage of SEM (Hitachi, Japan).

For hemocompatibility evaluation, 300 μL of red blood cell suspension was incubated with 300 μL of sample suspension (1 mg mL^−1^). After centrifugation, the absorbance of the supernatant at 545 nm was measured using a microplate reader. Deionized water and phosphate-buffered saline (PBS) served as positive and negative controls, respectively. Hemolysis rate (%) = (Sample absorbance – Negative control absorbance)/(Positive control absorbance – Negative control absorbance) × 100%.

For the swelling behavior measurement, HA-MIX and HA-MIX@N-SSAK with an initial weight (W_0_) were immersed in deionized water at pH 7.4 or pH 6.8 and incubated at 37 °C in a thermostatic shaker (Thermo Scientific, USA) at 100 rpm. At predetermined time points (0, 1, 3, 6, 12, 24, 36, 48, and 72 h), the hydrogels were removed, surface moisture was carefully absorbed, and the swollen weight (W_t_) was recorded. Swelling ratio (%) = (W_t_ − W_0_)/W_0_ × 100%.

The stability of the hydrogel was evaluated by assessing the degradation rate *in vitro* over time. The initial dry weight (W_0_) of the lyophilized hydrogel samples was first measured. The hydrogels were then immersed in PBS and incubated at 37 °C under gentle oscillation. At specified time intervals, the hydrogels were retrieved and lyophilized, with the resulting weight recorded as W_t_. The degradation rate was calculated as (W_0_ − W_t_)/W_0_ × 100%.

Rheological test of the HA-MIX@N-SSAK was conducted using a rotational rheometer (MCR92, Anton Paar). The strain and frequency were set at 1% and 1 Hz.

To assess self-healing capability, two identical hydrogels were individually dyed red and green, placed in contact within a petri dish, and the fusion time required for them to cohere without separation was recorded.

### 5.7 LC/MS

To identify the primary chemical constituents of T-SSAK and N-SSAK, an ultra-performance liquid chromatography coupled with quadrupole time-of-flight mass spectrometry (UPLC-Q/TOF-MS) system was employed. Separation was achieved using a Waters C18 column (2.1 mm × 100 mm, 1.7 μm) maintained at 35 °C, with a mobile phase flow rate of 0.3 mL min^−1^. The eluates were subsequently analyzed by tandem quadrupole time-of-flight mass spectrometry. All acquired data were processed and analyzed using UNIFI V1.8 software.

### 5.8 Animal experiments

Ten-week-old male C57BL/6J mice weighing 25-30 g were purchased from the Experimental Animal Center of Zhejiang Chinese Medical University (Hangzhou, China). The OA model was established through DMM surgery, while the sham surgery group (Sham) underwent only a joint capsule incision followed by suturing.

The therapeutic potential of different formulations was systematically evaluated through 3 consecutive experimental phases. Initially, the efficacy of T-SSAK was investigated. The animals were randomly divided into 4 experimental groups (n = 5 per group): Sham group, DMM group, T-SSAK (26 g kg^−1^ d^−1^) group, and Celecoxib (26 mg kg^−1^ d^−1^) group. Treatments were administered daily via oral gavage from postoperative day 2 through 8 weeks.

Subsequent evaluation was focused on N-SSAK efficacy. The mice were sorted into 4 groups (n = 5 per group): Sham group, DMM group, N-SSAK (0.8 g kg^−1^ d^−1^) group, and T-SSAK (26 g kg^−1^ d^−1^) group. The N-SSAK and T-SSAK doses were matched in terms of crude herbal material, based on the extraction yield of N-SSAK. The intervention regimen remained the same as before.

The final experimental phase evaluated the HA-MIX@N-SSAK Efficacy, comprising 5 groups: Sham group, DMM group, N-SSAK (4 mg/mL) group, HA-MIX hydrogel group, and HA-MIX@N-SSAK group. Each group was administered a 5-μL intra-articular injection of the respective preparation at 4-week intervals, with the first injection given post-surgery, and the study period spanning 8 weeks. Behavioral assessments were completed 24 h before harvesting tissue samples. All subjects were euthanized following the final intervention, and biological specimens were harvested for subsequent analytical procedures. All experimental procedures were approved by the Animal Ethics Committee of Zhejiang Chinese Medical University.

### 5.9 Pain-related behavioral and gait analyses

Mechanical sensitivity was quantified with von Frey filaments (0.008-300 g; Stoelting). A series of filament applications (5 s each), beginning at 0.16 g, were delivered to the right hind paw mid-plantar surface. The 50% withdrawal threshold was calculated via the Up-Down method.

Thermal pain sensitivity was evaluated using the Hargreaves test. Mice were placed on a YLS-6B intelligent hot plate apparatus (50.0 ± 0.5 °C, Mouse Specifics, USA), and the latency to licking or jumping of the right hind paw was recorded. Tests were conducted at 5-min intervals, and the mean of five measurements was calculated as the thermal pain threshold.

Gait analysis was performed using the DigiGait system (Mouse Specifics, USA). Mice ran on a motorized treadmill at a constant speed of 18 cm/s, and gait sequences of 6-10 s were recorded at 200 fps. The following parameters were analyzed: swing time (s), stance time (s), stride length (cm), and peak stance paw area (cm^2^).

Spontaneous activity was assessed using the open field test. The apparatus (40 × 40 × 40 cm PVC chamber) was cleaned with 75% ethanol prior to testing. Mice were placed in the central zone and allowed to explore freely for 30 min, with their movement trajectories recorded using an overhead camera. The data were processed using EthoVision XT 11 software by two trained observers blinded to experimental groupings.

### 5.10 Micro-CT analysis

Right knee joint specimens were fixed in 4% paraformaldehyde for 72 h. The samples were scanned using a high-resolution micro-CT (Skyscan 1176, Bruker μCT, Belgium). The region of interest (ROI) was delineated between the proximal tibial growth plate and the tibial plateau. A series of five consecutive sagittal images of the ROI was reconstructed three-dimensionally using visualization software (CTvol, SkyScan), and the BV/TV was calculated.

### 5.11 Histological analysis

After 14 days of decalcification in 14% EDTA solution, the right knee joint specimens were dehydrated using an automated tissue processor (Tissue-Tek VIP 5Jr, Japan) and embedded in paraffin. Serial sagittal sections of 4 μm thickness were prepared and subjected to ABH/OG staining and SO/FG staining to evaluate cartilage morphology and proteoglycan distribution. All tissue sections were digitally scanned using a whole-slide scanning system (3DHISTECH PANNORAMIC MIDI II, Hungary). Three observers experienced in orthopedic pathology performed a blinded semi-quantitative assessment of cartilage pathological changes according to the OARSI scoring system [69].

### 5.12 IHC and IF

Paraffin sections were baked at 60 °C for 4 h, followed by deparaffinization and rehydration. Antigen retrieval was performed in citrate buffer at 60 °C for 4 h. Sections were then permeabilized with Triton X-100 for 10 min and blocked with goat serum (1:20) at room temperature for 20 min. The sections were incubated overnight at 4 °C with the following primary antibodies: COL2 (1:200), Aggrecan (1:200), ADAMTS-5 (1:200), MMP-13 (1:300), GPX4 (1:200), ACSL4 (1:200), RBP4 (1:200), STRA6 (1:100), and CYP26B1 (1:100).

For IHC detection, after washing, sections were incubated with appropriate secondary antibodies at room temperature for 30 min, followed by DAB staining and hematoxylin counterstaining for 20 s. Digital images were acquired using a whole-slide scanner (3DHISTECH PANNORAMIC MIDI II, Hungary).

For IF detection, after primary antibody incubation, sections were incubated with Alexa Fluor® 488-conjugated anti-rabbit IgG (1:1000) at 37 °C for 40 min protected from light, followed by nuclear staining with DAPI (1:1000) for 10 min. Imaging was performed using a fluorescence microscope (LSM880, Zeiss, Germany). Quantitative analysis was conducted using ImageJ software.

### 5.13 In vivo retention of fluorescent labeling

To investigate the biodistribution of N-SSAK *in vivo*, near-infrared fluorescence labeling was performed using ICG. Mice were divided into three groups: PBS group, ICG (0.05 mg/mL) group, and ICG-labeled N-SSAK (ICG@N-SSAK) group. Animals were sacrificed at 6, 24, and 48 h post-administration. Major organs and tissues, including heart, liver, spleen, lung, kidney, bone, and gastrointestinal tract, were harvested and imaged using an *in vivo* imaging system (IVIS Spectrum, PerkinElmer, USA).

To further compare the retention capacity of different formulations within the joint cavity, fluorescently labeled N-SSAK, and HA-MIX@N-SSAK were injected into the joint cavities of mice, with fluorescence signal changes observed at different time points. Quantitative analysis of fluorescence intensity was performed using Living Image software.

### 5.14 Isolation and culture of primary cells

To obtain primary articular chondrocytes, 2-week-old mice were euthanized and disinfected by immersion in 75% ethanol for 10 min. Cartilage tissues were aseptically isolated from bilateral femoral heads, minced into fragments, and digested in DMEM/F-12 medium containing 0.25% collagenase D (11088858001, Merck, Germany) at 37 °C for 6-8 h. During digestion, gentle pipetting was performed hourly to facilitate cell dissociation. The cell suspension was filtered through a 70 μm cell strainer, centrifuged to collect cells, and resuspended in DMEM/F-12 complete medium supplemented with 10% fetal bovine serum and 1% penicillin-streptomycin for subsequent culture. To establish an *in vitro* model of osteoarthritic inflammation, the culture medium was replaced with serum-free DMEM/F-12 containing recombinant mouse IL-1β at a final concentration of 10 ng/mL.

### 5.15 CCK-8 assay

A CCK-8 kit was used to assess the cytotoxicity of N-SSAK on chondrocytes at various concentrations. Cells were seeded in 96-well plates at a density of 1 × 10^4^ cells per well, with peripheral wells filled with PBS to minimize evaporation. Blank control wells containing only medium were included for zero adjustment. After culturing for 12 h until the cell adhesion rate reached 70%-80%, N-SSAK solutions with final concentrations of 0.5, 1, 2, 4, 8, and 16 mg/mL were added to the wells, and the cells were further cultured in a 37 °C, 5% CO_2_ incubator for an additional 12 h. Following incubation, the medium was carefully removed and replaced with 100 μL of fresh medium containing 10 μL of CCK-8 reagent per well. After 1 h of incubation in the dark, the absorbance at 450 nm was measured using a microplate reader (Biotek Synergy, USA). Chondrocyte viability was calculated based on the optical density (OD) values obtained.

### 5.16 In vitro cellular uptake of N-SSAK

To assess the cellular uptake capacity of chondrocytes for N-SSAK, the nanoparticles were labeled with the red fluorescent dye DiD. The labeling process involved incubating the mixture at 37 °C in the dark for 30 min, followed by purification through ultracentrifugation at 100,000 ×g for 30 min at 4 °C. The labeled N-SSAK was then washed 3 times with PBS to obtain the final DiD-labeled N-SSAK. Chondrocytes were seeded at a density of 1 × 10^5^ cells per well, and the medium was replaced with serum-free medium containing DiD-labeled N-SSAK. After co-culturing at 37 °C for 3, 6, 12, and 24 h, the cell suspension was collected, centrifuged at 2000 rpm for 5 min, and resuspended in PBS for analysis of uptake efficiency using flow cytometry.

To investigate the internalization mechanism of N-SSAK, two experimental setups were established: one group involved co-culture of chondrocytes with 4 mg mL^−1^ DiD@N-SSAK for 24 h; another group was pretreated with 1 μmol L^−1^ chlorpromazine for 1 h prior to the addition of DiD@N-SSAK for 24 h. All samples were washed 3 times with PBS, fixed with 4% paraformaldehyde for 30 min, and subjected to nuclear staining with DAPI and cytoskeletal staining with FITC-phalloidin. Cellular internalization was visualized and imaged using fluorescence microscopy.

### 5.17 Micromass culture

To construct a chondrocyte microsphere model, primary chondrocytes were resuspended at a density of 1 × 10^7^ cells mL^−1^. A 20 μL aliquot of the cell suspension was carefully dropped in the center of a 12-well plate and incubated in a 37 °C incubator for 4–6 h to allow the cells to aggregate into spheres. Once the cells had formed aggregates, the medium was replaced with chondrogenic induction medium, consisting of DMEM/F12 as the base medium, supplemented with 10 ng/mL TGF-β3 (100-36E, Pepro Tech), 100 nmol/L dexamethasone (D1756, Sigma), 50 μg mL^−1^ L-ascorbic acid-2-phosphate (A8960, Sigma), and 1% ITS supplement (ITSS-10201-10, Cyagen). After appropriate induction culture, the cell microspheres were fixed with 4% paraformaldehyde for 30 min, washed 3 times with PBS, and then stained with 1% Alcian Blue (Sigma, A5268) for 30 min to evaluate the synthesis of glycosaminoglycans in the cartilage matrix.

### 5.18 RT-qPCR

Total RNA was extracted from chondrocytes using TRIzol reagent (Invitrogen, USA), with concentration and purity assessed by measuring absorbance at 260 nm and 280 nm. Reverse transcription was performed using a commercial kit (AQ11706, Accurate Biology) to synthesize cDNA. The mRNA expression levels of Col2, Aggrecan, MMP13, and ADAMTS5 were quantified using SYBR Premix Ex Taq kit (Takara, Japan) on an ABI 7500 Real-Time PCR System (Applied Biosystems, USA). β-actin was used as the reference gene, and the relative expression levels of each gene were calculated using the 2^−ΔΔCT^ method. All experiments were conducted independently in triplicate, and the primer sequences used are detailed in Table S5.

### 5.19 WB

After being washed 3 times with PBS, cells were lysed using RIPA buffer on ice for 30 min. The lysates were then scraped and centrifuged to collect the protein supernatant. Protein samples were separated by SDS-PAGE and transferred onto nitrocellulose membranes. The membranes were blocked at room temperature for 1 h, followed by incubation with primary antibodies at 4 °C overnight. The primary antibodies were as follows: GAPDH (1:5000), β-actin (1:5000), COL2 (1:1500), MMP-13 (1:1500), ADAMTS-5 (1:1000), Aggrecan (1:1000), GPX4 (1:1000), ACSL4 (1:1000), 4-HNE (1:1000), RBP4 (1:5000), STRA6 (1:1500), and CYP26B1 (1:1000). Subsequently, the membranes were incubated with HRP-conjugated secondary antibodies (1:5000) at room temperature for 1 h. Protein bands were visualized using a chemiluminescence imaging system (ChemiDoc XRS, BIO-RAD) and quantitatively analyzed with ImageJ software.

### 5.20 Analysis for GSH/GSSG and MDA

GSH and MDA levels were assessed using commercial assay kits. GSH content was determined according to the GSH and GSSG assay kit (S0053) protocol and measured colorimetrically at 412 nm. Lipid peroxidation was evaluated by MDA content using the lipid peroxidation assay kit and measured colorimetrically at 535 nm.

### 5.21 Analysis for ROS

ROS levels were assessed using a commercial ROS assay kit. Intracellular ROS in chondrocytes was detected by DCFH-DA (10 μmol/L, 30 min, 37 °C) staining and fluorescence microscopy, and tissue ROS in frozen joint sections was measured using the same procedure after rewarming.

### 5.22 Transcriptome analysis

Chondrocytes were divided into 3 experimental groups: control, IL-1β-stimulated, and IL-1β+N-SSAK groups. Total RNA was extracted from specifically treated chondrocytes (cell count >1 × 10^7^) using the TRIzol method. After quality control verification by NanoDrop (OD260/OD280 ratio = 1.8-2.0) and Agilent 2100 Bioanalyzer (RIN >8.0), qualified RNA samples were used to construct sequencing libraries with the NEBNext® Ultra™ RNA Library Prep Kit. The libraries were subjected to paired-end 150 bp sequencing on the Illumina NovaSeq 6000 platform. The bioinformatic analysis pipeline included: raw data quality control and filtering using Fastp, alignment of clean reads to the reference genome with HISAT2, transcript assembly and expression quantification using StringTie, identification of differentially expressed genes (DEGs) with DESeq2 (screening criteria: q < 0.05 and |log_2_FC| ≥ 1), followed by functional annotation of DEGs through GO analysis and pathway enrichment analysis using KEGG. GSEA-based pathway enrichment was conducted by ranking genes based on log_2_ fold change values and converting SYMBOL identifiers to ENTREZID using the org.Mm.eg.db annotation database. The gseKEGG function from the clusterProfiler package was employed for Gene Set Enrichment Analysis, with significant pathways selected using the following thresholds: P < 0.05, false discovery rate (FDR) < 0.25, and |NES| > 1. Enriched pathways were annotated with readable gene symbols via the DOSE package and visualized using the gseaplot2 function.

### 5.23 Statistical analysis

Statistical analysis and graph generation were performed using GraphPad Prism 9 software. All experimental data underwent normality and homogeneity of variance testing, with results reported as mean ± standard deviation. Comparisons between two groups were conducted using the independent samples *t*-test, while multiple group comparisons were analyzed by one-way ANOVA with Tukey's HSD test. A p-value of less than 0.05 was considered statistically significant.

## Ethics approval and consent to participate

All animal protocols in this study were approved by the Experimental Animal Ethics Committee of Zhejiang Chinese Medical University (20241104-16).

## CRediT authorship contribution statement

**Xuefeng Li:** Data curation, Formal analysis, Funding acquisition, Investigation, Project administration, Visualization, Writing – original draft, Writing – review & editing. **Yuhang Wang:** Data curation, Formal analysis, Investigation, Methodology, Visualization, Writing – original draft. **Xucheng Wang:** Data curation, Formal analysis, Investigation, Methodology, Visualization, Writing – original draft. **Wenzhe Chen:** Data curation, Formal analysis, Investigation, Methodology, Visualization. **Ling Jin:** Formal analysis, Investigation, Validation. **Qinwen Ge:** Formal analysis, Investigation, Validation. **Jingyuan Wen:** Formal analysis, Investigation, Validation. **Pinger Wang:** Data curation, Methodology, Validation. **Wenhua Yuan:** Data curation, Methodology, Validation. **Yimin Yang:** Data curation, Methodology, Validation. **Luwei Xiao:** Methodology, Supervision, Validation. **Jiali Chen:** Methodology, Supervision, Visualization, Writing – review & editing. **Di Chen:** Methodology, Supervision, Writing – review & editing. **Songfeng Hu:** Funding acquisition, Methodology, Project administration, Supervision. **Hongting Jin:** Conceptualization, Funding acquisition, Methodology, Project administration, Supervision, Visualization, Writing – review & editing.

## Declaration of competing interest

The authors declare that they have no known competing financial interests or personal relationships that could have appeared to influence.

## Data Availability

Data will be made available on request.
