# Supplementary material for: Self-assembled nanoparticles from natural herbs alleviate osteoarthritis by targeting retinol metabolism and inhibiting chondrocyte ferroptosis
Source: Mater Today Bio. 2026 May 17;38:103250. doi: 10.1016/j.mtbio.2026.103250 (PMC13214542; doi:10.1016/j.mtbio.2026.103250)
Supplement: Multimedia component 1 [file mmc1.docx]

**Fig. S1.** (A) Schematic diagram of animal behavior experiments. (B) Quantitative analysis of PWT. n = 5 per group. (C) Quantitative analysis of Hargreaves test. n = 5 per group. (D) Open field test of mice. n = 5. Values and error bars represent mean ± standard deviation. (*: P < 0.05, **: P < 0.01, ***: P < 0.001).

**Fig. S2.** LC-MS analysis of chemical constituents in T-SSAK. (A) Analysis in positive-ion mode. (B) Analysis in negative-ion mode.

**Fig. S3.** H&E staining of histological sections from major organs, including heart, liver, spleen, lung, and kidney. n = 3. Scale bar, 50 μm.

**Fig. S4.** (A) Tyndall effect and particle size distribution of N-SSAK at pH 1.2 over time. (B) Tyndall effect and particle size distribution of N-SSAK at pH 6.5 over time.

**Fig. S5.** (A) Quantitative analysis of PWT. n = 5 per group. (B) Quantitative analysis of Hargreaves test. n = 5 per group. (*: P < 0.05, **: P < 0.01, ***: P < 0.001).

**Fig. S6.** H&E staining of histological sections from major organs, including heart, liver, spleen, lung, and kidney. n = 3. Scale bar, 50 μm.

**Fig. S7.** (A) Changes in the viability of chondrocytes treated with different concentrations of N-SSAK for 48 h. (B) Changes in the viability of chondrocytes treated with different concentrations of Chlorpromazine for 24 h. Values and error bars represent mean ± standard deviation. (ns: no significance, *: P < 0.05, **: P < 0.01, ***: P < 0.001).

**Fig. S8.** (A) Heatmap of DEGs between the IL-1β group and IL-1β+N-SSAK group (unmarked, n=4). (B) Volcano plot showing the differential gene expression analysis between the IL-1β group and IL-1β+N-SSAK group. (C) PCA plot illustrating the distinct clustering of samples from the IL-1β group and IL-1β+N-SSAK group based on gene expression profiles. (D) GO enrichment analysis of DEGs between the two groups. (E) GSEA of DEGs between the IL-1β group and IL-1β+N-SSAK group. (F) Relative mRNA expression levels of CYP26A1of the chondrocytes in the the control, IL-1β, and IL-1β+N-SSAK groups. n = 3. (G) Representative IF images of ROS level in vitro. n = 5. Scale bar, 100 μm. (H) Quantitative analysis of ROS level. (I) Representative IF images of ROS level in vivo. n = 5. Scale bar, 100 μm. (J) Quantitative analysis of ROS level. (K) Quantitative analysis of MDA content. n = 3. Values and error bars represent mean ± standard deviation. (ns: no significance, *: P < 0.05, **: P < 0.01, ***: P < 0.001).

**Fig. S9.** (A) Representative IF images of Col2. n = 5. Scale bar, 20 μm. (B) Quantitative analysis of Col2 fluorescence area in each group. (C) Representative IF images of GPX4. n = 5. Scale bar, 20 μm. (D) Quantitative analysis of GPX4 fluorescence area in each group. (E) Representative IF images of ACSL4. n = 5. Scale bar, 20 μm. (F) Quantitative analysis of ACSL4 fluorescence area in each group.

**Fig. S10.** (A) Scanning electron micrographs of the HA-CHO and HA-ADH. (B) Demonstration of hydrogel adhesion to different biological tissues. (C) Visualization of the self-healing capability of hydrogels.

**Fig. S11.** (A) Quantitative analysis of PWT. n = 5 per group. (B) Quantitative analysis of Hargreaves test. n = 5 per group. Values and error bars represent mean ± standard deviation. (*: P < 0.05, **: P < 0.01, ***: P < 0.001).

**Fig. S12.** H&E staining of histological sections from major organs, including heart, liver, spleen, lung, and kidney. n = 3. Scale bar, 50 μm.

**Supplementary Tables**

**Table S1. Qualitative analysis of chemical components in T-SSAK (Positive)**

| **NO** | **RT (min)** | **Identification** | **Formula** | **M/Z** | **Mass Error (ppm)** |
| --- | --- | --- | --- | --- | --- |
| 1 | 0.81 | D-(+)-Sorbose | C_6_H_12_O_6_ | 203.0517 | -4.3 |
| 2 | 1.14 | Adenine nucleoside | C_10_H_13_N_5_O_4_ | 268.1036 | -1.7 |
| 3 | 4.25 | Paeonilactone-B | C_10_H_12_O_4_ | 197.0801 | -3.9 |
| 4 | 6.2 | Sinapyl alcohol_1 | C_11_H_14_O_4_ | 211.0957 | -3.9 |
| 5 | 7.73 | Isoliquiritin apioside | C_26_H_30_O_13_ | 551.178 | 3.8 |
| 6 | 8.01 | Corticosterone | C_21_H_30_O_4_ | 347.221 | -2 |
| 7 | 8.25 | Ligustilide | C_12_H_14_O_2_ | 191.1058 | -4.3 |
| 8 | 8.76 | Apigenin-7-O-neohesperidoside | C_27_H_30_O_14_ | 579.1718 | 1.6 |
| 9 | 8.83 | Senkyunolide F | C_12_H_14_O_3_ | 207.1007 | -4.3 |
| 10 | 9.26 | Syringetin | C_17_H_14_O_8_ | 347.0757 | -1.3 |
| 11 | 9.49 | Liquiritin | C_21_H_22_O_9_ | 419.1343 | 1.4 |
| 12 | 10.97 | Didymin | C_28_H_34_O_14_ | 595.2031 | 1.6 |
| 13 | 13.48 | Glabrolide | C_30_H_44_O_4_ | 469.3312 | 0 |
| 14 | 14.25 | Glyinflanin A | C_25_H_28_O_5_ | 431.1829 | 0.1 |
| 15 | 14.41 | Limonin | C_26_H_30_O_8_ | 471.2015 | 0.3 |
| 16 | 15.48 | Licoricesaponine J2 | C_42_H_64_O_16_ | 825.4283 | 1.9 |
| 17 | 15.88 | Isosakuranetin 7-rutinoside | C_22_H_24_O_9_ | 433.1492 | -0.4 |
| 18 | 16.3 | Isosinensetin | C20H20O7 | 373.1278 | -1 |
| 19 | 16.74 | Obacunone | C26H30O7 | 455.2062 | -0.6 |

**Table S2. Qualitative analysis of chemical components in T-SSAK (Negetive)**

| **NO** | **RT (min)** | | **Identification** | **Formula** | **M/Z** | **Mass Error (ppm)** |
| --- | --- | --- | --- | --- | --- | --- |
| 1 | 0.83 | Adipic acid | | C_6_H_10_O_4_ | 191.0561 | 0.1 |
| 2 | 1.09 | Citric acid | | C_6_H_8_O_7_ | 191.0198 | 0.6 |
| 3 | 1.59 | Aucubin_1 | | C_15_H_22_O_9_ | 391.1238 | -2.1 |
| 4 | 4.97 | 1-O-β-D-Glucopyranosylpaeonisuffrone | | C_16_H_24_O_9_ | 405.1409 | 1.7 |
| 5 | 6.17 | Citrusin A | | C_26_H_34_O_12_ | 583.2045 | 2.2 |
| 6 | 6.78 | Albiflorin R1 | | C_23_H_28_O_11_ | 525.1615 | 0.2 |
| 7 | 7.65 | Apigenin-7-O-neohesperidoside | | C_27_H_30_O_14_ | 577.1574 | 2 |
| 8 | 8.67 | Naringenin | | C_15_H_12_O_5_ | 271.0608 | -1.6 |
| 9 | 9.18 | Hesperetin 7-O-rutinoside | | C_28_H_34_O_15_ | 609.1839 | 2.3 |
| 10 | 9.61 | 4-O-Galloylalbiflorin | | C_30_H_32_O_15_ | 631.1693 | 3.9 |
| 11 | 10.96 | Plantamajoside | | C_29_H_36_O_16_ | 639.1948 | 2.8 |
| 12 | 12.65 | Licoricesaponine G2 | | C_42_H_62_O_17_ | 837.395 | 4.3 |
| 13 | 13.27 | Achybidensaponin I | | C_48_H_76_O_19_ | 955.4939 | 3.2 |
| 14 | 14.24 | Glyeurysaponin | | C_42_H_62_O_16_ | 821.4004 | 4.8 |
| 15 | 14.39 | Obaculactone | | C_26_H_30_O_8_ | 515.1936 | 2.6 |
| 16 | 16.86 | Isoastragaloside I | | C_45_H_72_O_16_ | 913.4834 | 3.5 |
| 17 | 17.94 | Achyranthoside IV | | C_41_H_60_O_15_ | 791.3897 | 4.7 |

**Table S3. Qualitative analysis of chemical components in N-SSAK (Positive)**

| **NO** | **RT (min)** | **Identification** | **Formula** | **M/Z** | **Mass Error (ppm)** |
| --- | --- | --- | --- | --- | --- |
| 1 | 1.25 | D-(+)-Sorbose | C_6_H_12_O_6_ | 203.0526 | 0.2 |
| 2 | 1.49 | Adenine nucleoside | C_10_H_13_N_5_O_4_ | 268.1042 | 0.7 |
| 3 | 6.38 | Sinapyl alcohol_1 | C_11_H_14_O_4_ | 211.0961 | -1.9 |
| 4 | 8.12 | Corticosterone | C_21_H_30_O_4_ | 347.2209 | -2.2 |
| 5 | 9.37 | Hesperetin 7-O-rutinoside | C_28_H_34_O_15_ | 611.1976 | 0.9 |
| 6 | 9.55 | Liquiritin | C_21_H_22_O_9_ | 419.1334 | -0.7 |
| 7 | 11.28 | Didymin | C_28_H_34_O_14_ | 595.2026 | 0.8 |
| 8 | 14.41 | Glyyunnanprosapogenin D | C_42_H_62_O_16_ | 823.4123 | 1.5 |
| 9 | 16.37 | Obacunone | C_26_H_30_O_7_ | 455.2061 | -0.6 |
| 10 | 17.58 | Ligustilide | C_12_H_14_O_2_ | 191.1061 | -3.1 |

**Table S4. Qualitative analysis of chemical components in N-SSAK (Negetive)**

| **NO** | **RT (min)** | **Identification** | **Formula** | **M/Z** | **Mass Error (ppm)** |
| --- | --- | --- | --- | --- | --- |
| 1 | 1.25 | Adipic acid | C_6_H_10_O_4_ | 191.0567 | 3.2 |
| 2 | 1.49 | Citric acid | C_6_H_8_O_7_ | 191.0204 | 3.5 |
| 3 | 2.87 | Geniposidic acid_1 | C_16_H_22_O_10_ | 373.1147 | 2 |
| 4 | 5.19 | 1-O-β-D-Glucopyranosylpaeonisuffrone | C_16_H_24_O_9_ | 405.1405 | 0.7 |
| 5 | 6.39 | Citrusin A | C_26_H_34_O_12_ | 583.2043 | 1.9 |
| 6 | 7.01 | Albiflorin R1 | C_23_H_28_O_11_ | 525.1626 | 2.3 |
| 7 | 8.12 | 4-O-Galloylalbiflorin | C_30_H_32_O_15_ | 631.168 | 1.9 |
| 8 | 8.79 | Apigenin-7-O-neohesperidoside | C_27_H_30_O_14_ | 577.1565 | 0.4 |
| 9 | 9.37 | Hesperetin 7-O-rutinoside | C_28_H_34_O_15_ | 609.1836 | 1.8 |
| 10 | 14.41 | Glyeurysaponin | C_42_H_62_O_16_ | 821.3985 | 2.4 |

**Table S5. The proportion of various compounds in the simulation system**

**(number of molecules)**

| **Number** | **Compound Name** | **Abbreviation** | **Relative content** | **1×Minimal** | **2×Minimal** |
| --- | --- | --- | --- | --- | --- |
| 1 | Corticosterone | COR | 4.76 | 5 | 10 |
| 2 | Hesperetin 7-O-rutinoside | HSR | 7.06 | 7 | 14 |
| 3 | Liquiritin | LIQ | 7.75 | 8 | 16 |
| 4 | Didymin | DID | 1.00 | 1 | 2 |
| 5 | Glyyunnanprosapogenin D | GYD | 2.28 | 2 | 5 |
| 6 | Obacunone | OBN | 10.39 | 10 | 21 |
| 7 | Citrusin A | CIT | 2.15 | 2 | 4 |
| 8 | Albiflorin R1 | ALR | 4.80 | 5 | 10 |
| 9 | 4-O-Galloylalbiflorin | GAB | 2.51 | 3 | 5 |
| 10 | Apigenin-7-O-neohesperidosid | APN | 4.81 | 5 | 10 |
| 11 | Glyeurysaponin | GYS | 3.18 | 3 | 6 |

**Table S6. RT–qPCR primers**

| **Primer** | **Primer sequence** **(5'to3')** | **Product length** |
| --- | --- | --- |
| Mouse GAPDH-F | GGTGAAGGTCGGTGTGAACG | 20 |
| Mouse GAPDH-R | CTCGCTCCTGGAAGATGGTG | 20 |
| Mouse GPX4-F | GATGGAGCCCATTCCTGAACC | 21 |
| Mouse GPX4-R | CCCTGTACTTATCCAGGCAGA | 21 |
| Mouse ACSL4-F | ATTGGTCAGGGATATGGGCT | 20 |
| Mouse ACSL4-R | AGAGGAGCTCCAACTCTTCCA | 21 |
| Mouse COL2-F | TGGTCCTCTGGGCATCTCAGGC | 22 |
| Mouse COL2-R | GGTGAACCTGCTGTTGCCCTCA | 22 |
| Mouse MMP13-F | TTTGAGAACACGGGGAAGA | 20 |
| Mouse MMP13-R | ACTTTGTTGCCAATTCCAGG | 20 |
| Mouse ADAMTS5-F | GGAGCGAGGCCATTTACAAC | 20 |
| Mouse ADAMTS5-R | CGTAGACAAGGTAGCCCACTTT | 22 |
| Mouse Aggrecan-F | GTGGAGCCGTGTTTCCAAG | 19 |
| Mouse Aggrecan-R | AGATGCTGTTGACTCGAACCT | 21 |
| Mouse RBP4-F | GGTGAGCAGCTTCAGAGTCA | 20 |
| Mouse RBP4-R  Mouse STRA6-F  Mouse STRA6-R  Mouse CYP26B1-F  Mouse CYP26B1-R  Mouse CYP26A1-F  Mouse CYP26A1-R  Mouse *β*-actin-F  Mouse *β*-actin-R | GCAAAAAGAGACCCTCGGGA  TGTCCTGCTCATTAACCTTCC  CAGACTATGGCTTGGCGAG  ACATCCACCGCAACAAGC  GGGCAGGTAGCTCTCAAGTG  CCGGCTTCAGGCTACAGA  GGAGCTCTGTTGACGATTGTT  GGAGATTACTGCCCTGGCTCCTA  GACTCATCGTACTCCTGCTTGCTG | 20  21  19  18  20  18  21  21  24 |
